# Tandem Cu(I)-Catalyzed Dipolar Cycloaddition–C–H Activation for the In-Flow Synthesis of *N*-Pyridyl-5-amino-1,2,3-triazole-4-carboxylates

**DOI:** 10.1021/acs.orglett.5c00453

**Published:** 2025-04-18

**Authors:** Emanuela Donato, Martha C. Mayorquín-Torres, Alessandra Puglisi, Maurizio Benaglia, Mauro F. A. Adamo, Christian V. Stevens

**Affiliations:** † Department of Green Chemistry and Technology, Ghent University, B-9000 Ghent, Belgium; ‡ Dipartimento di Chimica, Università degli Studi di Milano, 20133 Milano, Italy; § KelAda Pharmachem, Limited, A1.01 Science Centre South, Belfield, Dublin 4, Ireland; ∥ Centre for Synthesis and Chemical Biology, Department of Chemistry, Royal College of Surgeons in Ireland, 123 St Stephen’s Green, Dublin 2, Ireland

## Abstract

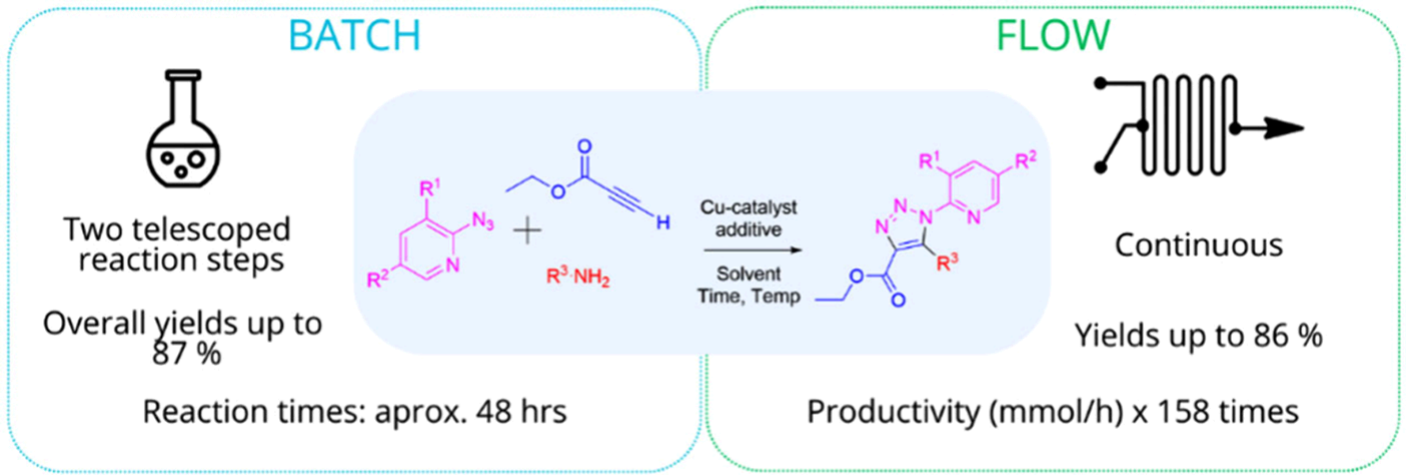

A telescoped process under continuous flow conditions is described for the synthesis of *N*-pyridyl-5-amino-1,2,3-triazole-4-carboxylate derivatives catalyzed by copper salts in a packed bed reactor. The synthetic approach takes first advantage of click chemistry, specifically relying on Cu(I)-catalyzed 1,3-dipolar azide–alkyne cycloaddition (CuAAC), to achieve the efficient and selective assembly of a triazole ring, followed by a copper-mediated C–H activation, that substitutes an inert C–H bond with a C–N bond, providing an environmentally acceptable and cost-effective strategy for synthesizing highly functionalized organic molecules.

In 2022, Professors Barry Sharpless, Morten Meldal, and Carolyn Bertozzi were awarded the Nobel Prize in Chemistry for their groundbreaking work on click reactions and biorthogonal transformations.^1^ Over the past 2 decades, “click chemistry”^2^ has become highly popular due to its high atom efficiency, rapid reaction rates, and robustness under various conditions, including the presence of oxygen and water.^3^ These innovations have greatly expanded the capabilities of synthetic and bioconjugate chemists, offering a versatile toolset for efficient chemical synthesis. Among several reactions that can be identified as “click reactions”, Huisgen 1,3-dipolar cycloaddition to obtain 1,2,3-triazoles particularly fits the definition.^4^

Moreover, the development of innovative techniques for directly converting C–H bonds to C–O, C–S, C–N, and C–C bonds remains an important goal in organic chemistry. The activation of inert C–H bonds represents the most significant challenge in C–H functionalization. Despite numerous studies in this subject, the concerns of reactivity and selectivity of C–H bonds prevent the wide applicability of this very valuable but challenging transformation.^5^ Recently, transition-metal-catalyzed C–H functionalization has emerged as an efficient and accessible synthetic method for the synthesis of a wide range of complex organic molecules.^6−8^ The published approaches demonstrated the importance of transition metals, such as Pd, Rh, Ru, and Ir, as catalysts in C–H functionalization.^9−12^ These metals were discovered to be extremely active in functionalizing C(sp^3^)–H, C(sp^2^)–H, and C(sp)–H bonds. However, the toxicity, poor abundance, and relatively high price of these metals slows down the widespread use of these catalysts.^13^

These disadvantages indicate the need to develop environmentally benign strategies for this type of reaction.^14,15^ Green chemistry prompted scientists to use less hazardous first row transition metals (such as Fe, Co, Ni, and Cu) as catalysts. Copper, for example, has received a lot of attention due to its low cost and abundance on Earth.^16^ One pioneer work about C–H activation using Cu for functionalization of aryl C–H bonds using O_2_ was reported by Yu and co-workers.^17^ The application of inexpensive Cu catalysts and O_2_ as the stoichiometric oxidant provides a considerable practical advantage. Moreover, the same researcher developed a copper(II)-mediated C–H amidation and amination reaction using a variety of sulfonamides, amides, and anilines. The amination reaction is extremely beneficial for the synthesis of medicinally relevant molecules.^18,19^

Chuprakov et al. reported an effective C5 arylation of 1,4-disubstituted 1,2,3-triazoles in good to excellent yields using palladium catalysis, tetrabutylammonium acetate (Bu_4_NOAc), and *N*-methylpyrrolidone (NMP) as the solvent. Furthermore, they demonstrated that this reaction is useful for the C5 regioselective arylation of 4,5-unsubstituted 1,2,3-triazoles (R_1_ = H), with the possibility of easily introducing aromatic electron-withdrawing (EW) substituents at the C5 position.^20^ Zhu et al. developed a Cu-catalyzed direct amination of 2-aryl-1,2,3-triazole *N*-oxides with primary and secondary amines.^21^

On the other hand, catalytic continuous flow processes are one of the most efficient, safe, and environmentally friendly techniques for producing active compounds. One strategy to perform efficient in-flow conversions is to use packed bed reactors.^22−25^

The aim of this study is to synthesize highly valuable compounds using a telescoped process under flow conditions. More specifically, using a packed-bed reactor and a copper salt as a catalyst, the *N*-pyridyl-1,2,3-triazole-4-carboxylate synthesis is developed under continuous flow conditions, followed by the in-flow introduction of an amino group by another packed-bed reactor filled with Cu(II) acetate (Scheme 1).

**Scheme 1:**
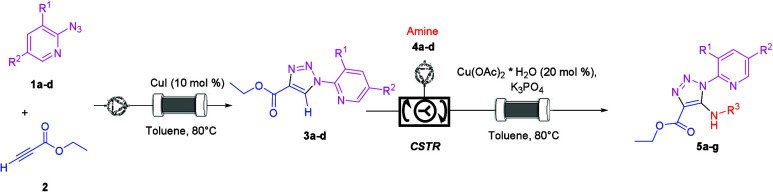
General Telescoped Process under Flow Conditions

First, the process under batch conditions was investigated, and all results obtained are reported in the Supporting Information. Subsequently, the click reaction and C–H activation were optimized under flow conditions.

The Vaportec easy-MedChem E-Series depicted in the Supporting Information was used for this transformation. A solution of the two reagents, azido pyridine **1a**–**1d** and ethyl propiolate **2**, in the appropriate solvent, was charged in an Erlenmeyer flask equipped with a stirring bar and placed onto a stirring plate. A tube connected to the peristaltic pump was inserted inside the reaction mixture. A packed-bed reactor was realized with an Omnifit column (10 mm/100 mm, 1× F, 1× A) containing CuI (0.1 equiv with respect to azido pyridine **1a**–**1d**) and sand (Scheme 1). The packed column was thermostated at 80 °C. The product was collected in vials for each residence time (*R*_t_) to evaluate the nuclear magnetic resonance (NMR) yield after solvent removal under reduced pressure. *R*_t_ was calculated experimentally passing solvent through the column using a flow rate of 1 mL/min. The product was purified by column chromatography using 7:3 dichloromethane (DCM)/EtOAc as the eluent (Scheme 2).^26^ The click reaction was optimized testing different flow rates and consequently different residence times (*R*_t_) (see the Supporting Information for all of the results). The best results using a flow rate of 0.100 mL/min are reported in Table 1, showing that yields in the range of 68–98% in dimethyl ether (DME) and the range of 89–93% in toluene were obtained. Productivity (*P*) and space–time yield (STY) were calculated for some selected results and are reported in Table 2.

**Scheme 2:**
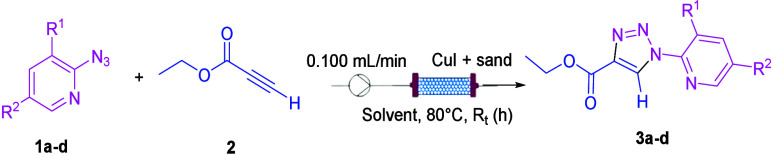
Click Reaction under Flow Conditions

**Table 1:** Click Reaction under Continuous Flow Conditions

entry	product	solvent	*R*_t_ (min)	isolated yield (%)
1	**3a**	DME	17	93
2	**3b**	DME	25	68
3	**3c**	DME	28	98
4	**3d**	DME	27	75
5	**3a**	toluene + DME	20	93
6	**3b**	toluene	20	91
7	**3c**	toluene	30	89
8	**3d**	toluene	30	93

**Table 2:** Productivity and STY Calculated for the Click Reaction

entry	**3a**–**3d**	solvent	*P*^a^ (mmol/h)	RF^b^	STY^c^ (mmol h^–1^ mL^–1^)	RF^d^
1	**3a**	DME	1.8	40	1.08	77
2	**3b**	DME	1.31	37	7.83 × 10^–1^	70
3	**3c**	DME	1.92	51	1.14	96
4	**3d**	DME	0.67	18	1 × 10^–1^	34
5	**3a**	toluene + DME	2.24	50	1.34	95
6	**3b**	toluene	1.77	50	1.06	95
7	**3c**	toluene	1.72	48	1.03	93
8	**3d**	toluene	1.92	51	1.15	97

a Productivity: moles of product (calculated from the isolated yield) divided by the collection time required to collect the product obtained by the reaction of 0.8 mmol of azido pyridine **1a**–**1d** (limiting agent of the telescoped process).

b Relative factor of productivity in flow vs batch (*P*_flow_/*P*_batch_).

c STY: moles of product in the reactor, divided by the residence time and reactor volume.

d Relative factor of STY in flow vs batch (STY_flow_/STY_batch_).

Productivities (mmol/h) of in-flow reactions were typically 18–51 times higher than those of in-batch transformations, while space time yields (mmol mL^–1^ h^–1^) for the continuous flow process were significantly higher, typically 34–97 times higher than those for the batch transformations. The first preliminary tests for the C–H activation were performed using Vaportec easy-MedChem E-Series, described in the Supporting Information.

The reaction was performed in toluene as solvent because Cu(OAc)_2_ and CuBr_2_ were partially soluble in DME (Scheme 3). CuBr_2_ was used as a catalyst, but after 1 h, clogging of the reactor occurred; therefore, CuSO_4_ was selected as a copper catalyst. The results reported in Table 3 were evaluated first by ^1^H NMR using 1,3,5-trimethoxybenzene as the standard. Moreover, the NMR yield was confirmed by isolation of the product using column chromatography.

**Scheme 3:**
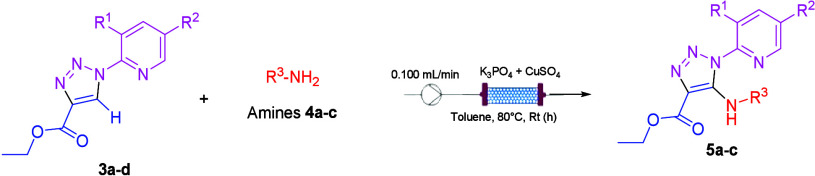
C–H Activation under Flow Conditions

**Table 3:** Productivity (mmol/h) and STY (mmol mL^–1^ h^–1^) for the Flow Process Using Vapourtec easy-MedChem E-Series

entry	**3**	**4**	*P*^a^ (mmol/h)	RF^b^	STY^c^ (mmol h^–1^ mL^–1^)	RF^d^
1	**3a**	**4a**	5.06 × 10^–1^	127	2.53 × 10^–1^	63
2	**3b**	**4b**	3.46 × 10^–1^	87	1.65 × 10^–1^	41
3	**3b**	**4c**	2.28 × 10^–1^	68	1.78 × 10^–1^	54

a Productivity: moles of product (calculated from the isolated yield) divided by the collection time required to collect the product obtained by the reaction of 0.2 mmol of triazoles **3a**–**3d** (limiting agent of the telescoped process).

b Relative factor of productivity in flow vs batch (*P*_flow_/*P*_batch_).

c STY: moles of product in the reactor, divided by the residence time and reactor volume.

d Relative factor of STY in flow vs batch (STY_flow_/STY_batch_).

A new set of experiments was performed using ASIA Syrris as a flow device and Cu(OAc)_2_˙H_2_O as the catalyst. The setup used is shown in the Supporting Information. The reaction mixture was pumped through the packed-bed reactor (Omifit, 10 mm/100 mm, 1× F, 1× A) containing K_3_PO_4_ and Cu(OAc)_2_·H_2_O, showed in the Supporting Information. The temperature of the packed bed reactor was set at 80 °C. The yields are in the range of 89–93% using toluene. Productivity (mmol/h) and STY (mmol h^–1^ mL^–1^) were calculated, and the results were reported in Table 4. The productivity increased 43–69 times compared to batch, and the STY increased 14–23 times.

**Table 4:**
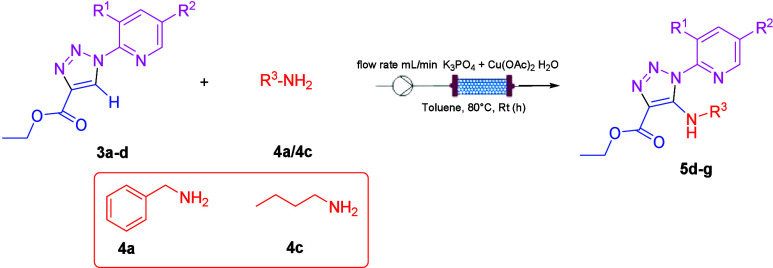
Productivity (mmol/h) and STY (mmol mL^–1^ h^–1^) for the Flow Process Using ASIA Syrris Premium

entry	R	*R*_t_ (min)	yield^a^ (%)	*P*^b^ (mmol/h)	RF^c^	STY^d^ (mmol h^–1^ mL^–1^)	RF^e^
1	**3b**	60	94	1.88 × 10^–1^	47	6.27 × 10^–2^	16
2	**3b**	30	92	3.68 × 10^–1^	69	1.23 × 10^–1^	23
3	**3d**	60	96	1.92 × 10^–1^	48	6.40 × 10^–2^	16
4	**3d**	45	91	2.43 × 10^–1^	46	8.09 × 10^–2^	15
5	**3c**	60	86	1.72 × 10^–1^	43	5.73 × 10^–2^	14

a All of the yields are isolated.

b Productivity: moles of product (calculated from the isolated yield) divided by the collection time required to collect the product obtained by the reaction of 0.2 mmol of triazoles **3b**–**3d** (limiting agent of the telescoped process).

c Relative factor of productivity in flow vs batch (*P*_flow_/*P*_batch_).

d STY: moles of product in the reactor, divided by the residence time and reactor volume.

e Relative factor of STY in flow vs batch (STY_flow_/STY_batch_).

Next, a telescoped process was developed under flow conditions (Scheme 4). The setup and procedure used for the telescoped process were described in the Supporting Information.

**Scheme 4:**
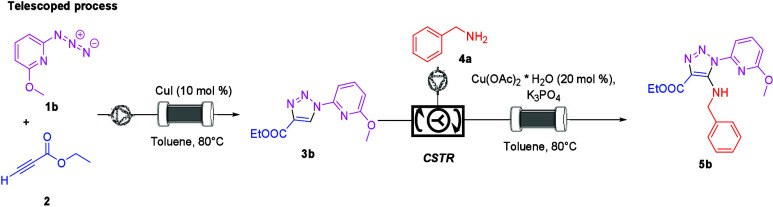
Telescoped Process: Synthesis of Aminotriazole **5b**

The telescoped flow process gave an excellent result. The overall yield of the process is 86%. In crude ^1^H NMR, only traces of the starting material **5b** were detected. Moreover, the productivity (mmol/h) is 158 times higher than that for the batch process, and a STY (mmol h^–1^ mL^–1^) is 79 times compared to the batch process (Table 5).

**Table 5:** Productivity (mmol/h) and STY (mmol mL^–1^ h^–1^) for the Telescoped Flow Process Using ASIA Syrris

entry	*R*_t_ (min)	overall yield (%)	*P*^a^ (mmol/h)	RF^b^	STY^c^ (mmol h^–1^ mL^–1^)	RF^d^
**1**	16	86	2.29	158	3.58 × 10^–1^	79

a Productivity: moles of product (calculated from the isolated yield) divided by the collection time required to collect the product obtained by the reaction of 0.8 mmol of azido pyridine **1a**–**1d** (limiting agent of the telescoped process).

b Relative factor of productivity in flow vs batch (*P*_flow_/*P*_batch_).

c STY: moles of product in the reactor, divided by the residence time and reactor volume.

d Relative factor of STY in flow vs batch (STY_flow_/STY_batch_).

In conclusion, the synthesis of *N*-pyridyl-5-alkylamino-1,2,3-triazole-4-carboxylate derivatives was performed in batch and under flow conditions, obtaining excellent results using different azido pyridines **1a**–**1d** and different amines **4a**–**4c**. Moreover, the telescoped process was performed for the synthesis of product **5b**, and an excellent result was obtained with an overall yield of 86%. The productivity (mmol/h) is 158 times higher that for the batch process, and the STY (mmol h^–1^ mL^–1^) is 79 times higher than that for the batch process.

## Data Availability

The data underlying this study are available in the published article and its online Supporting Information.

## Supporting Information Available

General procedure, experimental section, and product characterization (PDF)
