# Supplementary material for: Tandem Cu(I)-Catalyzed Dipolar Cycloaddition–C–H Activation for the In-Flow Synthesis of N-Pyridyl-5-amino-1,2,3-triazole-4-carboxylates
Source: Org Lett. 2025 Apr 18;27(17):4423–7. doi: 10.1021/acs.orglett.5c00453 (PMC12068680; doi:10.1021/acs.orglett.5c00453)
Supplement: Supplementary file 1 — ol5c00453_si_001.pdf [file ol5c00453_si_001.pdf]

ELECTRONIC SUPPORTING INFORMATION

A tandem Cu(I) catalysed dipolar cycloaddition - C–H activation for the in-flow synthesis of *N*-pyridyl-5-amino-1,2,3-triazole-4-carboxylates.

Emanuela Donato,[b] Martha C. Mayorquín-Torres,[a] Alessandra Puglisi,[b] Maurizio Benaglia,[b] Mauro F. A. Adamo,[c,d] Christian V. Stevens[a]*

[a] Department of Green Chemistry and Technology, Ghent University, B-9000 Ghent, Belgium. [b] Dipartimento di Chimica, Università degli Studi di Milano, Milano, Italy. [c] KelAda Pharmachem Ltd., A1.01. Science Centre South, Belfield, Dublin 4, Ireland. [d] Centre for Synthesis and Chemical Biology, Department of Chemistry, Royal College of Surgeons in Ireland, 123 St Stephen's Green, Dublin 2, Ireland.

Table of contents

1. General S2

Experimental Section S3

2.1 General procedure for the Click reaction under batch conditions S3

2.2 General procedure for the C−H activation under batch conditions S4

2.3 General procedure for the Click reaction under flow conditions S5

2.3.1 Optimization of the Click reaction under flow conditions S6

2.4 General procedure for the C−H activation under flow conditions S9

2.5 General procedure for telescoped process under flow conditions S9

2.6 General Set Up under flow condition for Click reactions S10

2.7 General Set Up under flow condition for C−H functionalization. S10

2.8 Set Up under flow condition for telescoped synthesis S11

3 Product characterisation S12

3.1 ^{1}H, ^{13}C NMR spectra S15

1. General

- Reactions were monitored by glass-backed silica plates (Merck Silicagel 60 F254, precoated, thickness 0.25 mm) in combination with an appropriate solvent mixture. All compounds could be visualized by UV irradiation (254 or 365 nm).
- Flash chromatography was carried out on silica gel (230-400 mesh).
- Routine ^{1}H-NMR and ^{19}F-NMR analysis were recorded on a benchtop NMR, Spinsolve Multi X, 80 MHz, Magriteck). Characterizations were recorded on Brucker Avance 300 spectrometer, operating at 300 MHz for ^{1}H-NMR and 75 MHz for ^{13}C-NMR, with complete proton decoupling. Brucker Avance 400 spectrometer, operating at 400 MHz for ^{1}H-NMR and 101 MHz for ^{13}C-NMR, with complete proton decoupling. All NMR spectra were recorded using the standard pulse sequences and parameters recommended by the manufacturer and were processed employing the MestreNova NMR processing program.
- Proton chemical shifts are reported in ppm (δ) with the solvent reference relative to tetramethylsilane (TMS) employed as the internal standard ($CDCl_3$ $\delta = 7.26$ ppm). Carbon chemical shifts are reported in ppm (δ) relative to TMS with the respective solvent resonance as the internal standard ($CDCl_3$, $\delta = 77.0$ ppm). The following abbreviations are used to indicate the multiplicity in NMR spectra: s - singlet; d - doublet; t - triplet; q - quartet; pd – pseudo doublet; dd - double doublet; sext – sextuplet; sept – septuplet; bs - broad signal; m - multiplet.
- HPLC: For HPLC analyses an Agilent Instrument Series 1100 was used.
- Mass spectra: Mass spectra were registered on an APEX II & Xmass software (Bruker Daltonics) instrument or on a thermo Finnigan LCQ Advantage instrument, equipped with an ESI-TOF ion source. COSPECT mass spectroscopy (University of Milan).
- Melting points (mp) were registered using Stuart Scientific, Mealting Point apparatus SMP3. Temperature resolution: 0.1°C, Sensor: PT100. Ramp rate used 1 °C/min.
- Azido compounds are highly explosive and toxic and thus must be handled with extreme caution.

Experimental Section

2.1 General procedure for the Click reaction under batch conditions

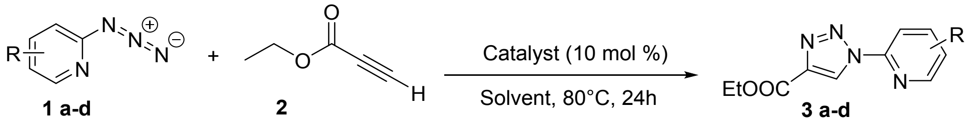

Scheme 1. Cycloaddition reaction under batch conditions

Procedure A: To a solution of 2-azidopyridine **1a-d** (99-120 mg, 1 mmol, 1 equiv.) in dry solvent (0.25 M), copper acetate (18.2 mg, 0.1 mmol, 0.1 equiv.) and sodium ascorbate (0.05 mmol, 0.05 equiv.) were added, followed by ethyl propiolate (**2**) (107.9 mg, 1.1 mmol, 1.1 equiv.). The reaction mixture was stirred for 24 hours in an oil bath at 80°C, in a sealed vial, until complete consumption of the starting materials, monitored by TLC (DCM/AcOEt 7:3), to obtain **3a-d**.

Procedure B: To a solution of 2-azidopyridine **1a-d** (99-120 mg, 1 mmol, 1 equiv.) in dry solvent (0.25 M), copper iodine (19.0 mg, 0.1 mmol, 0.1 equiv.) were added, followed by ethyl propiolate (**2**) (107.9 mg, 1.1 mmol, 1.1 equiv.). The reaction mixture was stirred for 24 hours in an oil bath at 80°C in a sealed vial, until complete consumption of the starting materials, monitored by TLC (DCM/AcOEt 7:3), to obtain **3a-d**.

2.1.1 Optimization for batch conditions

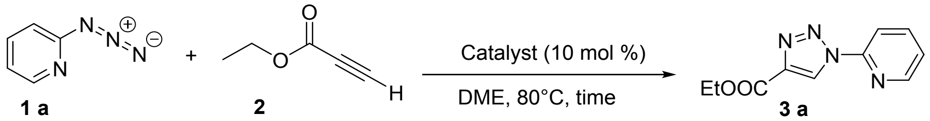

Scheme 2. Cycloaddition reaction between the azido pyridine **1a-d** and ethyl propiolate **2**

Table 1. Optimization click reaction under batch conditions in DME

Entry	Catalyst	Na Ascorbate (equiv.)	Time (h)	^{1}H-NMR Yield (%)
**1**	$CuSO_4 \cdot 5H_2O$	0.1 eq.	20	87
**2**	$Cu(OAc)_2$	0.05 eq.	16	89
**3**	CuI	-	16	98

Different copper salts were used. The reaction was heated in an oil bath at 80°C in a closed vial. Different reaction times and heating devices were tested. Firstly, the click reaction was studied using the model substrate **1a** and different copper salts were tested. The yields for compound **3a** are reported in Table 1. It is noteworthy that the method using Cu(I) allows the production of **3a** in high yield without using sodium ascorbate.

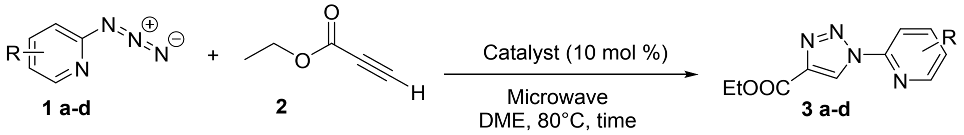

Scheme 3. Cycloaddition reaction between azido pyridine (**1a-d**) and propiolate (**2**) tested using microwave irradiation.

Table 2. Results obtained for the click reaction using microwave in DME.

Entry	Product	Time (h)	Catalyst	Sodium Ascorbate (equiv.)	Yield (%)
**1**	**3a**	1	$CuSO_4{\cdot}5H_2O$	0.1 eq.	88
**2**		1	$Cu(OAc)_2$	0.05 eq.	89
**3**		1	CuI	-	92
**4**	**3b**	1	$Cu(OAc)_2$	0.05 eq.	89
**5**		1.5	CuI	-	79
**6**	**3c**	1.5	CuI	-	89
**7**	**3d**	1.5	CuI	-	85

It is interesting to highlight that heating the cycloaddition reaction in a microwave reduces reaction times while affording high yields and full conversion of azido pyridine **1a-d**. The results are reported in Table 2.

2.2 General procedure for the C−H activation under batch conditions

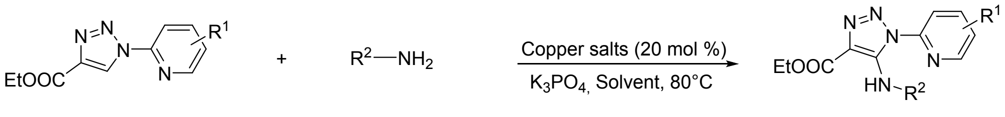

Scheme 4. General procedure for the C−H activation under batch conditions.

To a solution of triazol **3a-d** (99-120 mg, 1 mmol, 1 equiv.,), copper salt (25-35 mg, 0.2 mmol, 0.2 equiv.) and potassium phosphate (424.5 mg, 2 mmol, 2 equiv.) in solvent (0.2 M), amine **4** (80-150 mg, 3 mmol, 3 equiv.) was added. The reaction was set up in an oil bath at 80°C in a sealed vial due to the solvent low boiling point (bp=85°C). After 24 hours, TLC (DCM/AcOEt 7:3) showed complete consumption of the starting materials. Once cooled to room temperature, each crude was washed with DCM/H_2O three times, the organic phases collected and dried over Na_2SO_4. Hence, the solvent was removed by rotary evaporation and further purification occurred via column chromatography to afford the product **5a-g**.

After the studies on the click reaction in batch, the C−H activation was also studied under different experimental conditions (Scheme 5). Model substrate **3b** was tested using different amines **4a-c** and different copper sources in the presence of K_3PO_4 as a base (Table 3).

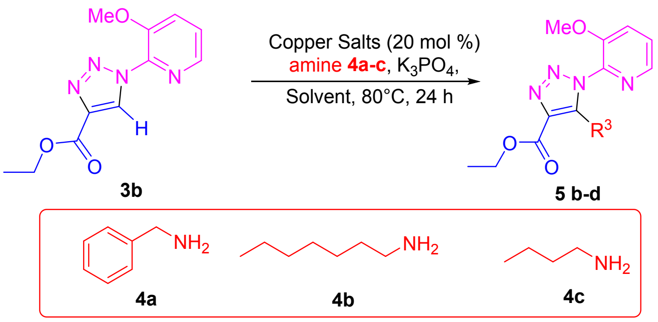

Scheme 5. C-H activation under batch conditions using compound 3b and amines 4a-c.

Table 3. C-H activation using different copper salts.

Entry	Amine	Catalyst	^{1}H-NMR Yield (%)[a]
1	**4a**	$Cu(OAc)_2$	/
2	**4a**	$Cu(OAc)_2{\cdot}H_2O$	94 (90)
3	**4a**	$CuBr_2$	78 (46)
4	**4b**	$Cu(OAc)_2$	/

5	**4b**	$CuBr_2$	69 (70)
**6**	**4c**	$Cu(OAc)_2{\cdot}H_2O$	95 (96)
**7**	**4c**	$CuBr_2$	/

[a] Yields between brackets are isolated yields. The NMR yield was calculated using 1,3,5-trimethoxybenzene as an internal standard.

The reaction worked well with all the amines and the copper salts. In particular, using $Cu(OAc)_2{\cdot}H_2O$ excellent yields were achieved using benzylamine (**4a**) and butylamine (**4c**). The scope of the reaction was further investigated, by combining *N*-pyridyl triazoles **3c-d** and amines **4a-d**, using $Cu(OAc)_2{\cdot}H_2O$ as a catalyst, K_3PO_4 as a base and DME or toluene as a solvent. The results are reported in Table 4.

Table 4. C-H activation reaction scope with **amines 4a-4d.**

**Entry**	**Solvent**	**R**	**amine**	**Isolated Yield (%)**
**1**	DME	F	**4a**	94
**2**	DME	F	**4c**	92
**3**	DME	Me	**4a**	83
**4**	DME	Me	**4c**	48[a]
**5**	DME	Me	**4d**	27[a]
**6**	Toluene	F	**4a**	92
**7**	Toluene	F	**4c**	87
**8**	Toluene	Me	**4a**	76
**9**	Toluene	Me	**4c**	43[a]
**10**	Toluene	Me	**4d**	18[a]

[a] NMR yield

When R is a methyl group which is an Electron Donating Group (EDG), the yield is 48% while in presence of fluorine in meta position, which is an Electron-Withdrawing Group (EWG) the yield goes up to 92%. The reaction was tested in DME, which is considered the best solvent for this kind of transformation, but also in toluene which was previously demonstrated as a good solvent for click reactions. In toluene, the reaction shows a similar trend using an EWG while in the presence of an EDG the yield drops to 43 %. The methyl group in meta position of the pyridine makes the yield to drop in DME, but also in toluene, while the yield is comparable using DME or toluene having a fluorine in meta position of the pyridine.

2.3 General procedure for the Click reaction under flow conditions

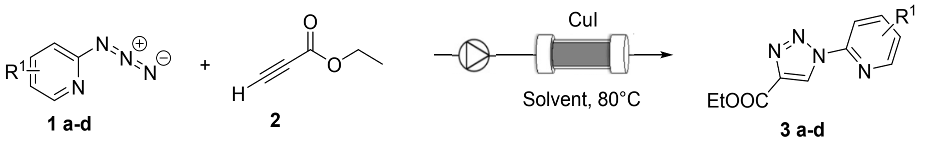

Scheme 6. General procedure for the Click reaction under flow conditions.

The reaction mixture, azido pyridine **1a-d** (10 mmol, 1 equiv.) and ethyl propionate (**2**) (1.07 g 11 mmol, 1.1 equiv.) were put in an Erlenmeyer with a stirring bar under nitrogen atmosphere. A tube connected to the peristaltic pump was insert inside the reaction mixture. Moreover, another tube related to the packed column, (Omnifit 10MM/100MM 1xF1xA). The packed column was heated at 80°C using the Vapourtec. The column was filled with a mixture of CuI (190 mg, 1 mmol, 0.1 equiv.) and sand. At the end, the product was collected in vials for each residence time (R_t) to evaluate the NMR yield. The Rt was calculated experimentally passing through the column solvent using a flow rate of 1 mL/min. In conclusion, internal standard 1,3,5-trimethoxybenzene was added and the solvent was removed by rotary evaporation to evaluate the NMR yield. The product was purified by chromatography (DCM: EtOAc 7:3).

2.3.1 Optimization of the Click reaction under flow conditions

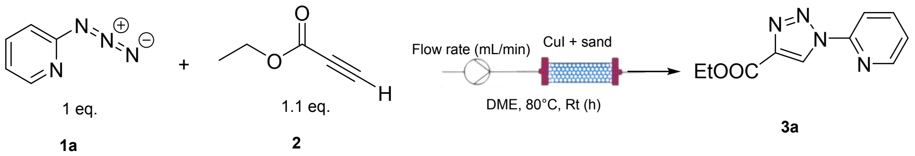

Scheme 7. Cycloaddition reaction between the azido pyridine **1a** and ethyl propiolate (**2**) in flow.

Table 5. Optimization click reaction under flow condition.

Entry	Flow (mL/min)	R_t(min)	^{1}H-NMR Yield(%)	P[a] (mmol/h)	RF[b]	STY[c] (mmol/h*mL)	RF[d]
**1**	0.100	17	93	1.8	40	1.08	77
**2**	0.250	10	49	2.39	53	1.43	102
**3**	0.500	5	3	$2.68\ 10^{-1}$	0.2	$1.61\text{x}10^{-1}$	0.8

^{1}H-NMR yield was calculated using as internal standard 1,3,5-trimethoxybenzene. [a] Productivity: moles of product (calculated from NMR yield) divided by the collection time required to collect the product obtained by reaction of 0.8 mmol of azido pyridine (limiting agent). [b] Relative factor of productivity in flow vs in batch (P_{flow}/P_{batch}). [c] STY: moles of product in reactor, divided by residence time and reactor volume. [d] Relative Factor of STY in flow vs in batch (STY_{flow}/STY_{batch}).

Productivities (mmol/h) of in-flow reactions were typically 40-53 times higher than in batch transformations, while space time yields (mmol/ml*h) for the continuous flow process were significantly higher (typically 77–102 times higher) (**Table 5**). It is interesting to highlight that the productivity and the Space Time Yield are better in entry 2, table 3 but in that case, there is no complete consumption of the azido pyridine **1a**, while in entry 1 using a flow rate of 0.100 mL/min, the complete conversion of the azido pyridine **1a** was achieved.

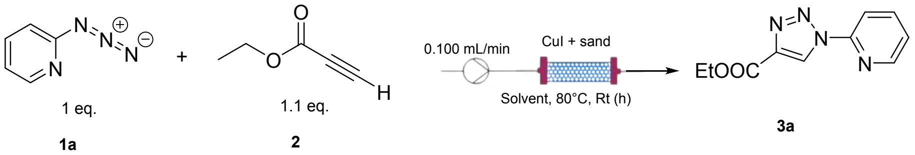

Scheme 8. Cycloaddition reaction between the azido pyridine **1a** and ethyl propiolate (**2**) in flow with different solvents.

For further investigation we set a flow rate of 0.100 mL/min and we screened different solvents. The results are reported in **Table 6**. The azido pyridine **1a** was not completely soluble in toluene, therefore 10% of DME was added in the reaction mixture.

Table 6. Optimization of the cycloaddition reaction in flow conditions using different solvents.

Entry	Solvent	R_t (min)	^{1}H-NMR Yield (%)	P[a] (mmol/h)	RF[c]	STY[c] (mmol/h*mL)	RF[d]
**1**	Toluene + DME (9:1)	20	93	2.24	50	1.34	95
**2**	Cyrene	30	94	1.50	33	$9.01\text{x}10^{-1}$	64
**3**	DMF	17	91	2.59	57	1.55	110

The NMR yield was calculated using an internal standard, 1,3,5-trimethoxybenzene. [a] Productivity: moles of product (calculated from NMR yield) divided by the collection time required to collect the product obtained by reaction of 0.8 mmol of azido pyridine (limiting agent). [b] Relative factor of productivity in flow vs in batch (P_{flow}/P_{batch}). [c] STY: moles of product in reactor, divided by residence time and reactor volume. [d] Relative Factor of STY in flow vs in batch (STY_{flow}/STY_{batch}).

In all solvents tested, the reaction worked with good results in terms of productivity and space time yield. The productivity increased in the range of 33-50 times compared to batch and the STY increased in a range of 64-110 times compared to batch. The 2-Me-THF was not tested in flow conditions since the catalyst is partially soluble. Toluene was the best solvent tested as compared to Cyrene and DMF, since it is easy to remove after the process because it has a boiling point of 110°C. Moreover, the same click reaction was tested using the azido pyridine **1b** under flow conditions. Also, in this case the column was packed with CuI and sand. The reaction was again optimized using different flow rates and corresponding residence times. The results are reported in **Table 7.**

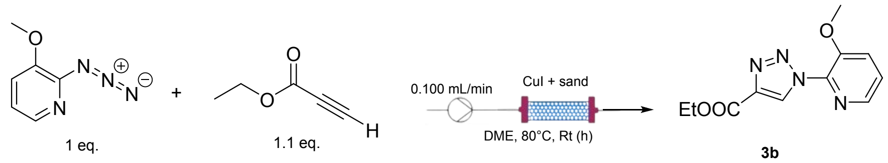

1b 2 3b

Scheme 9. Cycloaddition reaction between the azido pyridine **1b** and ethyl propiolate **2** in flow.

Table 7. Optimization of the reaction conditions for the formation of the product **1b**

Entry	Flow (mL/min)	Rt(min)	^{1}H-NMR Yield(%)	P[a] (mmol/h)	RF[b]	STY[c] (mmol/h*mL)	RF[d]
**1**	0.100	25	68	1.31	37	7.83x10^{-1}	70
**2**	0.250	10	24	1.14	32	32	61
**3**	0.500	5	2	2.20x10^{-1}	0.2	1.31x10^{-1}	0.9

The NMR yield was calculated using as internal standard 1,3,5-trimethoxybenzene. [a] Productivity: moles of product (calculated from NMR yield) divided by the collection time required to collect the product obtained by reaction of 0.8 mmol of azido pyridine (limiting agent). [b] Relative factor of productivity in flow vs in batch (P_{flow}/P_{batch}). [c] STY: moles of product in reactor, divided by residence time and reactor volume. [d] Relative Factor of STY in flow vs in batch (STY_{flow}/STY_{batch}).

Productivities (mmol/h) of in-flow reactions were typically 32-37 times higher than in batch transformations, while space time yields (mmol/ml*h) for the continuous flow process were significantly higher, typically 61–70 times higher (**Table 7**).

In all solvents tested, the reaction worked with good results in terms of productivity and space time yield. The productivity increased in the range of 36-50 times compared to batch and the STY increased in the range of 68-95 times compared to batch. Toluene was the best solvent tested as compared to Cyrene and DMF. The yield obtained is the highest and it is easy to remove after the reaction because has a boiling point of 110°C compared to DMF and Cyrene (**Table 8**).

Table 8. Optimization of the cycloaddition reaction in flow conditions using different solvents.

Entry	Solvent	Rt (min)	^{1}H-NMR Yield (%)	P[a] (mmol/h)	RF[c]	STY[c] (mmol/h*mL)	RF[d]
**1**	Toluen e	20	91	1.77	50	1.06	95
**2**	Cyrene	30	87	1.69	47	1.43	91
**3**	DMF	17	66	1.27	36	7.60x10^{-1}	68

The NMR yield was calculated using as internal standard 1,3,5-trimethoxybenzene. a) Productivity: moles of product (calculated from NMR yield) divided by the collection time required to collect the product obtained by reaction of 0.8 mmol of azido pyridine (limiting agent). b) Relative factor of productivity in flow vs in batch (P_{flow}/P_{batch}). c) STY: moles of product in reactor, divided by residence time and reactor volume. d) Relative Factor of STY in flow vs in batch (STY_{flow}/STY_{batch})

Subsequently, using the azido pyridine **1c** the reaction was tested only using a flow rate of 0.100 mL/min. The reaction was tested only in DME and toluene and not in other solvents because it was observed previously that these are the two best solvent for this transformation. The results are reported in **Table 9**.

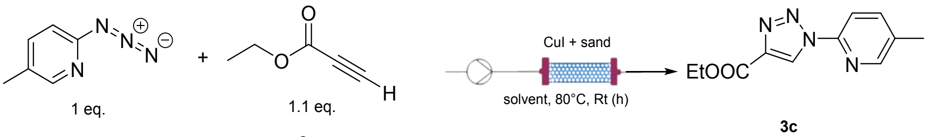

1c **2** **3c**

Scheme 10. Cycloaddition reaction between the azido pyridine **1c** and ethyl propiolate **2** in flow.

Table 9. Optimization of the reaction conditions for the formation of the product **3c**

Entry	Solvent	Rt (min)	^{1}H-NMR Yield (%)	P^{a} (mmol/h)	RF^{c}	STY^{c} (mmol/h*mL)	RF^{d}
**1**	DME	28	98	1.92	51	1.14	96
**2**	Toluene	30	89	1.72	48	1.03	93

The NMR yield was calculated using as internal standard 1,3,5-trimethoxybenzene. [a] Productivity: moles of product (calculated from NMR yield) divided by the collection time required to collect the product obtained by reaction of 0.8 mmol of azido pyridine (limiting agent). [b] Relative factor of productivity in flow vs in batch (P_{flow}/P_{batch}). [c] STY: moles of product in reactor, divided by residence time and reactor volume. [d] Relative Factor of STY in flow vs in batch (STY_{flow}/STY_{batch}).

The productivity in DME increased 51 times compared to the batch condition and the STY increased 96 times, while in toluene the productivity having a residence time of 0.5 h is 48 times higher compared to batch and the STY is 93 times higher than the batch conditions. The results are reported in **Table 9**.

In conclusion, the click reaction was performed also using the azido pyridine **1d.** The reaction was tested using the optimized condition only in DME and Toluene. The reaction performed in DME gave moderate results obtaining 75 % yield and not full conversion of starting materials, while in toluene the reaction gave 93% yield.

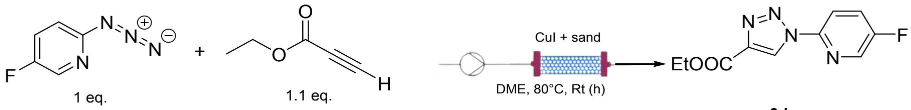

1d **2** **3d**

Scheme 11. Cycloaddition reaction between the azido pyridine **1d** and ethyl propiolate (**2**) in flow.

Table 10. Optimization of the reaction conditions for the formation of the product **3d**

Entry	Solvent	Rt (min)	^{1}H-NMR Yield (%)	P^{a} (mmol/h)	RF^{c}	STY^{c} (mmol/h*mL)	RF^{d}
**1**	DME	27	75	$6.7x10^{-1}$	18	$1x10^{-1}$	34
**2**	Toluene	30	93	1.92	51	1.15	97

The NMR yield was calculated using as internal standard 1,3,5-trimethoxybenzene. [a] Productivity: moles of product (calculated from NMR yield) divided by the collection time required to collect the product obtained by reaction of 0.8 mmol of azido pyridine (limiting agent). [b] Relative factor of productivity in flow vs in batch (P_{flow}/P_{batch}). [c] STY: moles of product in reactor, divided by residence time and reactor volume. [d] Relative Factor of STY in flow vs in batch (STY_{flow}/STY_{batch}).

The productivity (mmol/h) in DME increased 18 times compared to batch conditions and the STY (mmol/h*mL) increased 34 times, while in toluene the productivity (mmol/h) is 51 times and the STY (mmol/h*mL) is 97 times higher than in batch. The results are reported in **Table 10**.

2.4 General procedure for the C−H activation under flow conditions

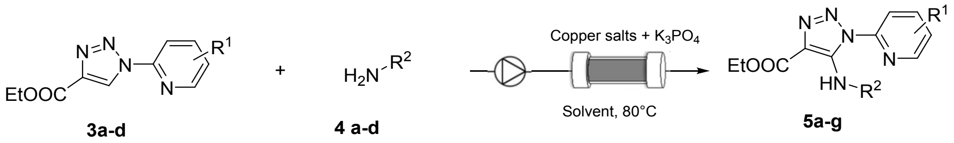

Scheme 12. General procedure for the C−H activation under flow conditions.

A solution of triazol **3a-d** (180-230 mg, 1 equiv., 3 mmol) and amine **4a-d** (300-600 µL, 3 equiv., 9 mmol) in Toluene was put in an Erlenmeyer with a stirring bar, the catalyst and closed with reverse cup, for avoiding the evaporation of the amine. Subsequently, the Ominift reactor was filled with K_3PO_4 firstly (1.3 g, 2 equiv. 6 mmol) separated by a layer of sand and followed by the copper salts (30-60 mg, 0.1 equiv., 0.3 mmol). The solution was passed through the column. The column was heated at 80° using the Vapourtec. The R_t was calculated experimentally passing through the column solvent using a flow rate of 1 mL/min. The set up is shown in Figure 1. In the end, the product was collected in vials for each residence time (R_t) to evaluate the NMR yield. After the reaction, the solvent was removed by rotary evaporation and further purification occurred via column chromatography (DCM: EtOAc 7:3).

2.5 General procedure for telescoped process under flow conditions

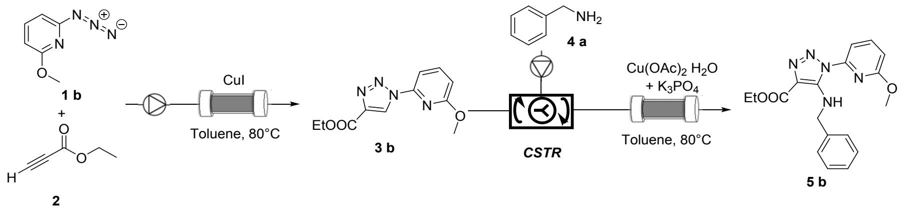

Scheme 13. Procedure telescoped process under flow conditions.

A solution of azido pyridine **1b** (1.50 g, 10 mmol. 1 equiv.) and ethyl propionate (**2**) (1.08 g, 11 mmol, 1.1 equiv.) in toluene under N_2 atmosphere (Mixture A) with a flow rate of 50 µL/min. A packed bed reactor was filled using CuI (1 mmol, 0.1 equiv. 190.1 mg) catalyst and sand. The flask with the mixture A related to the first packed bed reactor (Omnifit 10MM/100MM 1xF1xA). The first packed bed reactor was connected to a Continuous Stirred-Tank Reactor CSTR reactor. A flask with the amine (3.21g, 30 mmol, 3 equiv.), flow rate 50 µL/min, was put under N_2 and connected (Mixture B) at the CSTR The CSTR was connected to another packed bed reactor filled with $Cu(OAc)_2$ H_2O (399.5 mg, 2 mmol, 0.2 equiv.) and K_3PO_4 (4.24 mg,20 mmol, 2 equiv.). The total flow rate is 100 µL/min. Both columns were thermostated at 80°C. All the solution was collected and isolated using flash column chromatography (DCM:EtOAc 7:3) obtaining 3.04 g, 86 % overall yield.

2.6 General Set Up under flow condition for Click reactions

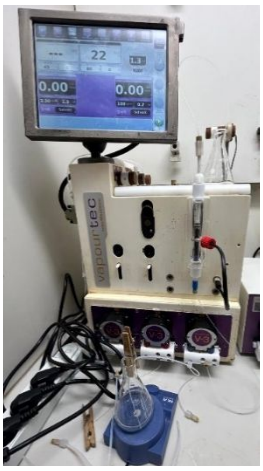

Figure 1. Vapourtec easy Med Chem E-Series

The Vapourtec easy Med Chem E-Series depicted in Figure 1 was used. A solution of the two reagents, azido pyridine **1a-d** (990mg-1.5 g, 10 mmol, 1 equiv.) and ethyl propiolate 2 (1.07 mg, 1.1 mmol, 1.1 equiv.), in the appropriate solvent, was charged in an Erlenmeyer flask equipped with a stirring bar and placed onto a stirring plate. A tube connected to the peristaltic pump was inserted inside the reaction mixture. A packed-bed reactor was realized with an Omnifit column (10MM/100MM 1xF1xA) containing CuI (190.1 mg, 1 mmol 0.1 equiv.) and sand. The packed column was thermostated at 80°C. The product was collected in vials for each residence time (R_t) to evaluate the NMR yield after solvent removal under reduced pressure. The R_t was calculated experimentally passing solvent through the column using a flow rate of 1 mL/min. The product was purified by column chromatography using DCM: EtOAc 7:3 as eluent.

2.7 General Set Up under flow condition for C−H functionalization.

- **Set Up 1**: depicted in Figure 1.
- **Set up 2**: Asia Premium Syrris depicted in Figure 2. The reaction mixture was pumped through the packed-bed reactor (Omifit 10MM/100MM 1xF1xA) containing K_3PO_4 (1.3 g, 6 mmol, 2 equiv.) and $Cu(OAc)_2 \cdot H_2O$. (119.7 mg, 0.2 mmol, 0.2 equiv.) The temperature of the packed bed reactor was set at 80 °C.

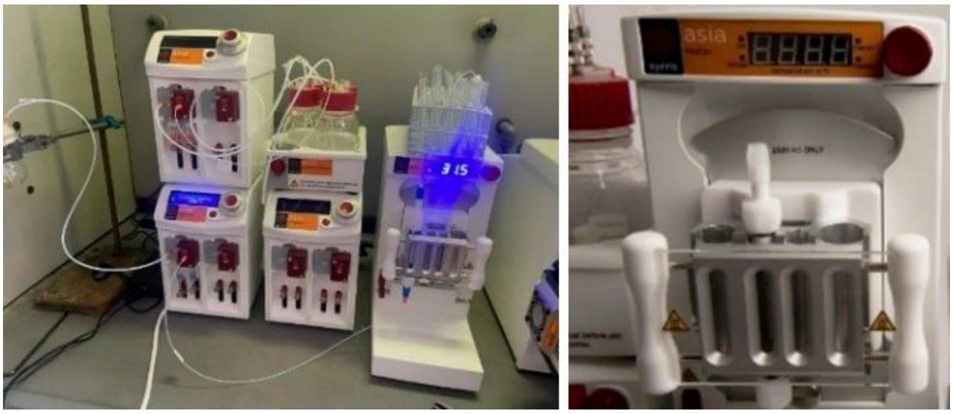

Figure 2. Set up using ASIA Premium Syrris

2.8 Set Up under flow condition for telescoped synthesis

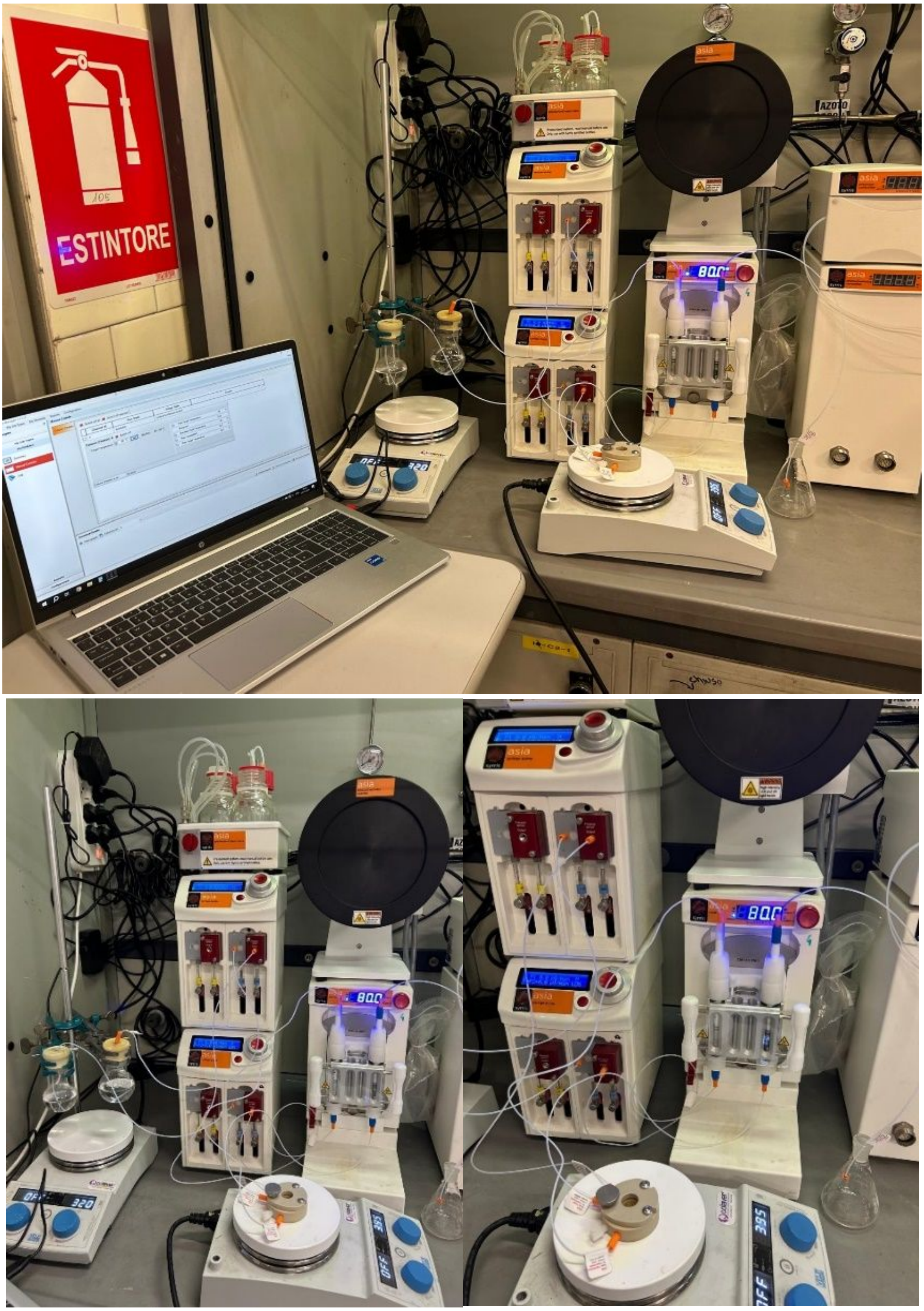

Figure 3. Set Up under flow condition for the telescoped process using ASIA Premium Syrris.

3 Product characterisation

- **Ethyl 1-(pyridin-2-yl)-1H-1,2,3-triazole-4-carboxylate (3a)**

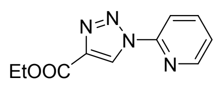

3a

yellow/orange solid. 93% NMR yield batch process, 200.5 mg 92% yield isolated yield by column chromatography (DCM:EtOAc 7:3) batch process, 98% NMR yield flow process, 1.39 g 91% isolated yield by column chromatography (DCM:EtOAc 7:3) flow process. mp: 105.1 -106.6 °C (DCM: EtOAc 7:3)

^{1}H NMR (400 MHz, $CDCl_3$): δ 9.09 (s, 1H), 8.53-8.55 (d, J = 4.7 Hz, 1H), 7.49-7.98 (t, *J* = 7.8 Hz, 1H), 7.40-7.43 (dd, *J* = 7.3, 5.0 Hz, 1H), 4.45-4.50 (q, *J* = 7.1 Hz, 2H), 1.43-1.45 (t, *J* = 7.1 Hz, 3H).

^{13}C NMR (101 MHz, $CDCl_3$) δ 160.6, 148.8, 148.6, 140.5, 139.5, 124.8, 124.3, 114.1, 61.4, 14.3.

HRMS (ESI) m/z [M] + Calcd. for $C_{10}H_{11}N_4O_2$: 218,0804 found; 218,0806.

- **Ethyl 1-(3-methoxypyridin-2-yl)-1H-1,2,3-triazole-4-carboxylate (3b)**

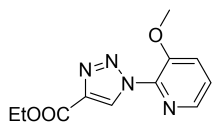

3b

Brown solid. 93% NMR yield batch process, 220.9 mg, 89% yield isolated yield by column chromatography (DCM:EtOAc 7:3) batch process,86% NMR yield flow process, 1.41 g 80% isolated yield by column chromatography (DCM:EtOAc 7:3) flow process. mp: 94.5-95.8 °C (from DCM/EtOAc). ^{1}H NMR (400 MHz, $CDCl_3$) δ 8.73 (s, 1H), 8.26 (d, *J* = 4.4 Hz, 1H), 7.53 – 7.46 (m, 2H), 4.45-4.51 (q, *J* = 7.1 Hz, 2H), 3.97 (s, 3H), 1.26-1.46 (t, J = 7.1 Hz, 3H).

^{13}C NMR (101 MHz, $CDCl_3$) δ 160.8, 148.0, 140.4, 139.6, 138.0, 128.8, 126.0, 121.3, 61.3, 56.4, 14.3.

HRMS (ESI) m/z [M]+ Calcd. for $C_{11}H_{11}N_4O_3$ 248,0909; found: 248,0914.

- **Ethyl 1-(5-methylpyridin-2-yl)-1H-1,2,3-triazole-4-carboxylate (3c)**

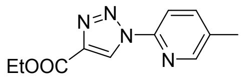

3c

Dark orange solid. 94% NMR yield batch process, 213.5 mg, 92% yield isolated yield by column chromatography (DCM:EtOAc 7:3) batch process, 99% NMR yield flow process,1.5 g, 92% isolated yield by column chromatography (DCM:EtOAc 7:3) flow process. mp: 100.7 -102.8 °C, (from DCM/EtOAc) ^{1}H NMR (400 MHz, $CDCl_3$) δ 9.04 (s, 1H), 8.34 (s, 1H), 8.13 (d, *J* = 8.3 Hz, 1H), 7.74-7.76 (d, *J* = 8.2 Hz, 1H), 4.44-4.50 (q, *J* = 7.1 Hz, 2H), 2.43 (s, 3H), 1.42-1.46 (t, *J* = 7.1 Hz, 3H).

^{13}C NMR (101 MHz, $CDCl_3$) δ 160.6, 148.8, 146.6, 140.4, 139.7, 134.5, 124.6, 113.6, 61.4, 18.1, 14.3.

HRMS (ESI) m/z [M]+ Calcd. for $C_{11}H_{12}N_4O_2$ 232,0960, found 232,0963.

- **Ethyl 1-(5-fluoropyridin-2-yl)-1H-1,2,3-triazole-4-carboxylate (3d)**

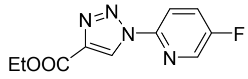

3d

Dark brown solid. 88 % NMR yield batch process, 210.1 mg, 89 % yield isolated yield by column chromatography (DCM:EtOAc 7:3) batch process, 96% NMR yield flow process, 1.55 g 94% isolated yield by column chromatography (DCM:EtOAc 7:3) flow process. mp: 95.3-98.7 °C (from DCM/EtOAc) ^{1}H NMR (400 MHz, $CDCl_3$) δ 9.04 (s, 1H), 8.41 (s, 1H), 8.27-8.30 (d, *J* = 8.3 Hz, 1H), 7.68-7.73 (d, *J* = 8.2 Hz, 1H), 4.46-4.52 (q, *J* = 7.1 Hz, 2H), 1.44-1.48 (t, *J* = 7.1 Hz, 3H).

^{13}C NMR (101 MHz, $CDCl_3$) δ 160.5, 158.1, 144.6, 140.7, 136.9, 126.5, 124.8, 115.5, 61.5, 14.3.

^{19}F NMR (376 MHz, $CDCl_3$): -125.40-125.58.

HRMS (ESI) m/z [M]+ Calcd. for $C_{10}H_{10}FN_4O_3$. 236,0710 found: 236,0713.

- **Ethyl 5-(benzylamino)-1-(pyridin-2-yl)-1H-1,2,3-triazole-4-carboxylate (5a)**

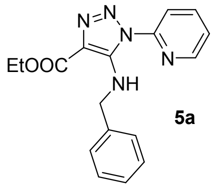

5a

Yellow solid. 258.6 mg, 80% Isolated yield by column chromatography (DCM:EtOAc 7:3) batch process. 85% NMR yield flow process, 853.1 mg 88% isolated yield by column chromatography (DCM:EtOAc 7:3) flow process. mp: 82.4-83.7 °C (from DCM/EtOAc), (from DCM/EtOAc).

^{1}H NMR (400 MHz, $CDCl_3$) δ 11.67 (s, 1H), 10.38 (d, *J* = 6.4 Hz, 1H), 8.05 (m, 1H), 7.56 (m, 1H), 7.36 (d, *J* = 4.9 Hz, 4H), 7.29 (m, 1H), 6.85 – 6.75 (m, 2H), 4.37 (d, *J* = 5.5 Hz, 2H), 4.17 (s, 1H), 4.11 (q, *J* = 7.1 Hz, 2H), 1.27 (t, *J* = 7.1 Hz, 3H).

^{13}C NMR (101 MHz, $CDCl_3$) δ 171.6, 158.9, 155.0, 145.9, 138.2, 128.7, 127.1, 116.4, 112.8, 64.3, 58.4, 46.1, 14.7.

HRMS (ESI) m/z [M +H]+ Calcd. for $C_{17}H_{18}FN_5O_2$ 323,1382, found: 324,1356.

- **Ethyl 5-(benzylamino)-1-(3-methoxypyridin-2-yl)-1H-1,2,3-triazole-4-carboxylate (5b)**

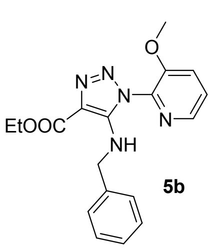

5b

Yellow solid. 94% NMR yield batch process, 317.9 mg, 90% isolated yield by column chromatography (DCM:EtOAc 7:3) batch process. 94% NMR yield flow process, 634.8 mg 90% isolated yield by column chromatography (DCM:EtOAc 7:3) flow process. mp: 114.2 -116.3 °C, (from DCM/EtOAc). ^{1}H NMR (400 MHz, $CDCl_3$) δ 11.78 (s, 1H), 10.47 (s, 0H), 7.63 (dd, *J* = 5.1, 1.4 Hz, 1H), 7.43 – 7.24 (m, 6H), 7.05 (dd, *J* = 8.0, 1.5 Hz, 1H), 6.78 (dd, *J* = 8.0, 5.1 Hz, 1H), 4.38 (d, *J* = 5.5 Hz, 2H), 4.20 (s, 1H), 4.14 (q, *J* = 7.1 Hz, 2H), 3.97 (s, 3H), 1.26 (t, *J* = 7.1 Hz, 3H).

^{13}C NMR (101 MHz, $CDCl_3$) δ 171.3, 158.5, 146.3, 143.5, 137.8, 136.4, 128.7, 127.3, 127.1, 115.9, 64.9, 58.3, 55.9, 46.1, 29.7, 14.7.

HRMS (ESI) m/z [M +Na] + Calcd. for $C_{18}H_{19}N_5O_3Na$ 376,1488 found: 376,2567.

- **Ethyl 5-(heptylamino)-1-(3-methoxypyridin-2-yl)-1H-1,2,3-triazole-4-carboxylate (5c)**

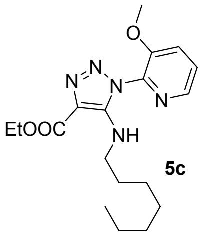

5c

Brown solid. 69% NMR yield batch process, 252.9 mg, 70 % isolated yield by column chromatography (DCM:EtOAc 7:3) batch process. 69 % NMR yield flow process, 505.3 mg 70% isolated yield by column chromatography (DCM:EtOAc 7:3) flow process. mp: 99.7-100.8 °C (from DCM/EtOAc). ^{1}H NMR (400 MHz, $CDCl_3$) δ 11.72 (s, 1H), 9.98 (d, *J* = 5.3 Hz, 1H), 7.68 (dd, *J* = 5.1, 1.4 Hz, 1H), 7.04 (dd, *J* = 8.0, 1.4 Hz, 1H), 6.79 (dd, *J* = 8.0, 5.1 Hz, 1H), 4.21 – 4.11 (m, 3H), 3.96 (s, 3H), 1.67 (dd, J = 22.5, 15.3 Hz, 2H), 1.47 – 1.23 (m, 11H), 0.93 – 0.85 (m, 4H).

^{13}C NMR (101 MHz, $CDCl_3$) δ 171.3, 158.4, 146.3, 143.5, 136.4, 115.8, 63.9, 58.2, 55.9, 42.1, 31.4, 29.7, 28.9, 28.6, 27.0, 22.6, 14.8, 14.1

HRMS (ESI) m/z [M +H] + Calcd. for $C_{18}H_{28}N_5O_3$ 361,2114 found: 362,2167

- **Ethyl 5-(butylamino)-1-(3-methoxypyridin-2-yl)-1H-1,2,3-triazole-4-carboxylate (5d)**

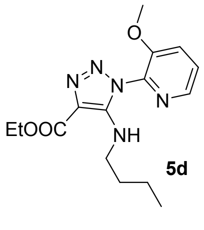

5d

Brown solid. 95% NMR yield batch process, 346.9 mg, 96 % isolated yield by column chromatography (DCM:EtOAc 7:3) batch process. 91% NMR yield flow process, 1.18 g 92% isolated yield by column chromatography (DCM:EtOAc 7:3). mp: 116.2 -117.3 °C (from EtOH) ^{1}H NMR (300 MHz, $CDCl_3$) δ 11.71 (s, 1H), 9.99 (d, *J* = 5.5 Hz, 1H), 7.67 (dd, *J* = 5.1, 1.6 Hz, 1H), 7.02 (dd, *J* = 8.0, 1.5 Hz, 1H), 6.77 (m, 1H), 4.15 (m, 3H), 3.93 (s, *J* = 1.3 Hz, 3H), 3.17 – 3.05 (m, 2H), 1.65 (p, *J* = 7.2 Hz, 2H), 1.43 (h, *J* = 7.3 Hz, 2H), 1.33 – 1.22 (m, 3H), 1.00 – 0.89 (m, 3H).

^{13}C NMR (75 MHz, $CDCl_3$) δ 171.2, 158.3, 146.2, 143.4, 136.3, 115.7, 63.9, 58.1, 55.9, 41.7, 30.7, 20.2, 14.8, 13.7.

HRMS (ESI) m/z [M +H] + Calcd. for $C_{15}H_{20}N_5O_3$ 319,1644 found 320,1656.

- **Ethyl 5-(benzylamino)-1-(5-fluoropyridin-2-yl)-1H-1,2,3-triazole-4-carboxylate (5f)**

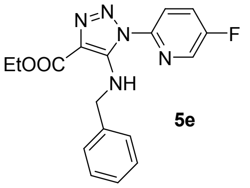

5e

Brown solid. 316.8 mg, 94 % isolated yield by column chromatography (DCM:EtOAc 7:3) batch process. 87% NMR yield flow process, 931.5 mg 91% isolated yield by column chromatography DCM:EtOAc 7:3) flow process. mp:118.7 -119.6 °C (from DCM/EtOAc). ^{1}H NMR (300 MHz, $CDCl_3$) δ 11.79 (s, 1H), 9.97 (d, *J* = 5.7 Hz, 1H), 7.94 (d, *J* = 3.0 Hz, 1H), 7.38 (d, *J* = 6.6 Hz, 4H), 6.80 (dd, *J* = 9.1, 3.6 Hz, 1H), 4.37 (d, *J* = 5.4 Hz, 2H), 4.22 – 4.06 (m, 3H), 1.29 (t, *J* = 7.1 Hz, 3H).

^{13}C NMR (75 MHz, $CDCl_3$) δ 171.7, 158.5, 153.1, 151.50, 137.5, 133.0, 132.6, 128.7, 127.4, 127.0, 126.6, 126.3, 113.7, 64.3, 58.5, 46.1, 14.7.

^{19}F NMR (276 MHz, $CDCl_3$) δ -137.54

HRMS (ESI) m/z [M +H] + Calcd. for $C_{17}H_{19}FN_5O_2$ 341,1288 found 342,1296

- **Ethyl 5-(butylamino)-1-(5-fluoropyridin-2-yl)-1H-1,2,3-triazole-4-carboxylate (5e)**

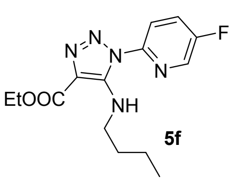

5f

Brown solid. 283.2 mg, 92 % isolated yield by column chromatography (DCM:EtOAc 7:3) batch process. 89% NMR yield flow process, 589.2 mg 87% isolated yield by column chromatography (DCM:EtOAc 7:3) flow process. mp:110.4 -111.9 °C (from EtOH). ^{1}H NMR (300 MHz, $CDCl_3$) δ 11.71 (s, 1H), 9.45 (t, *J* = 5.3 Hz, 1H), 7.95 (d, *J* = 3.0 Hz, 1H), 7.33 (m, 1H), 6.74 (dd, *J* = 9.1, 3.6 Hz, 1H), 4.12 (d, *J* = 6.4 Hz, 3H), 3.10 (q, *J* = 6.4 Hz, 2H), 1.64 (p, *J* = 7.2 Hz, 2H), 1.43 (m, 2H), 1.28 (t, *J* = 7.1 Hz, 3H), 0.95 (t, *J* = 7.3 Hz, 3H).

^{13}C NMR (75 MHz, $CDCl_3$) δ 171.6, 158.4, 156.3, 152.9,132.8, 126.4, 113.7, 63.4, 58.3, 41.7, 30.7, 20.2, 14.7, 13.6.

^{19}F NMR (276 MHz, $CDCl_3$) δ -137.38.

HRMS (ESI) m/z [M +Na] + Calcd. for $C_{14}H_{19}FN_5O_2Na$ 330,1445 found: 330,1464.

- **Ethyl 5-(benzylamino)-1-(5-methylpyridin-2-yl)-1H-1,2,3-triazole-4-carboxylate (5g)**

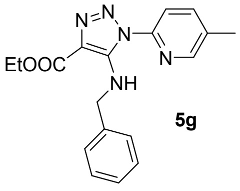

5g

Brown solid. 279.9 mg, 83% isolated yield by column chromatography (DCM:EtOAc 7:3) batch process. 84% NMR yield flow process, 580.3 mg, 86% isolated yield by column chromatography (DCM:EtOAc 7:3) flow process. mp: 105.6-107.1 °C (from DCM/EtOAc). ^{1}H NMR (300 MHz, $CDCl_3$) δ 11.59 (s, 1H), 10.39 (t, *J* = 5.6 Hz, 1H), 7.88 (d, *J* = 2.3 Hz, 1H), 7.47 – 7.23 (m, 6H), 6.72 (d, *J* = 8.4 Hz, 1H), 4.37 (d, *J* = 5.5 Hz, 2H), 4.20 – 4.06 (m, 3H), 2.22 (s, 3H), 1.28 (t, *J* = 7.1 Hz, 3H).

^{13}C NMR (75 MHz, $CDCl_3$) δ 171.6, 159.0, 152.9, 145.4, 139.2, 137.7, 128.7, 127.3, 127.0, 125.6, 112.3, 63.9, 58.3, 46.1, 17.5, 14.8.

HRMS (ESI) m/z [M +H] + Calcd. for $C_{18}H_{19}N_5O_2$ 337,1539 found 338,1543.

3.1 ¹H, ¹³C NMR spectra

- **¹H NMR (400 MHz, $CDCl_3$) of ethyl 1-(pyridin-2-yl)-1H-1,2,3-triazole-4-carboxylate (3a)**

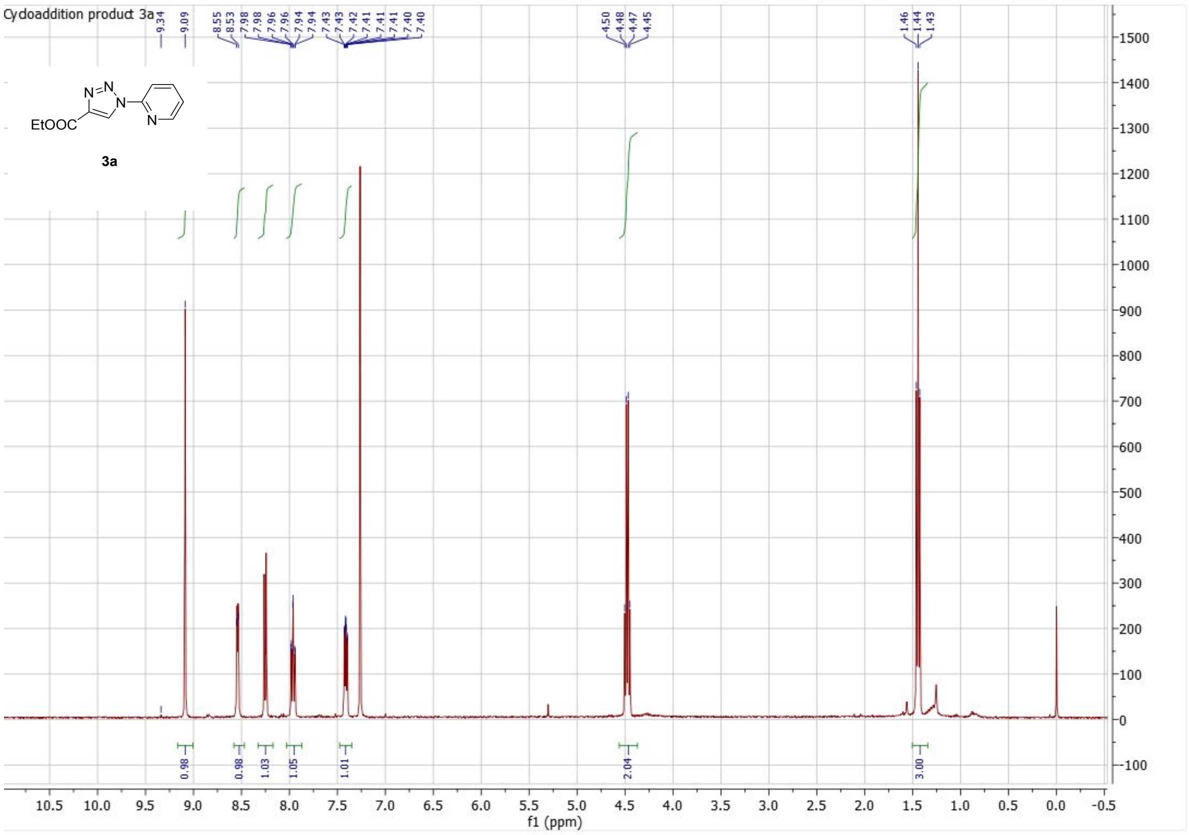

- **¹³C NMR (101 MHz, $CDCl_3$) of ethyl 1-(pyridin-2-yl)-1H-1,2,3-triazole-4-carboxylate (3a)**

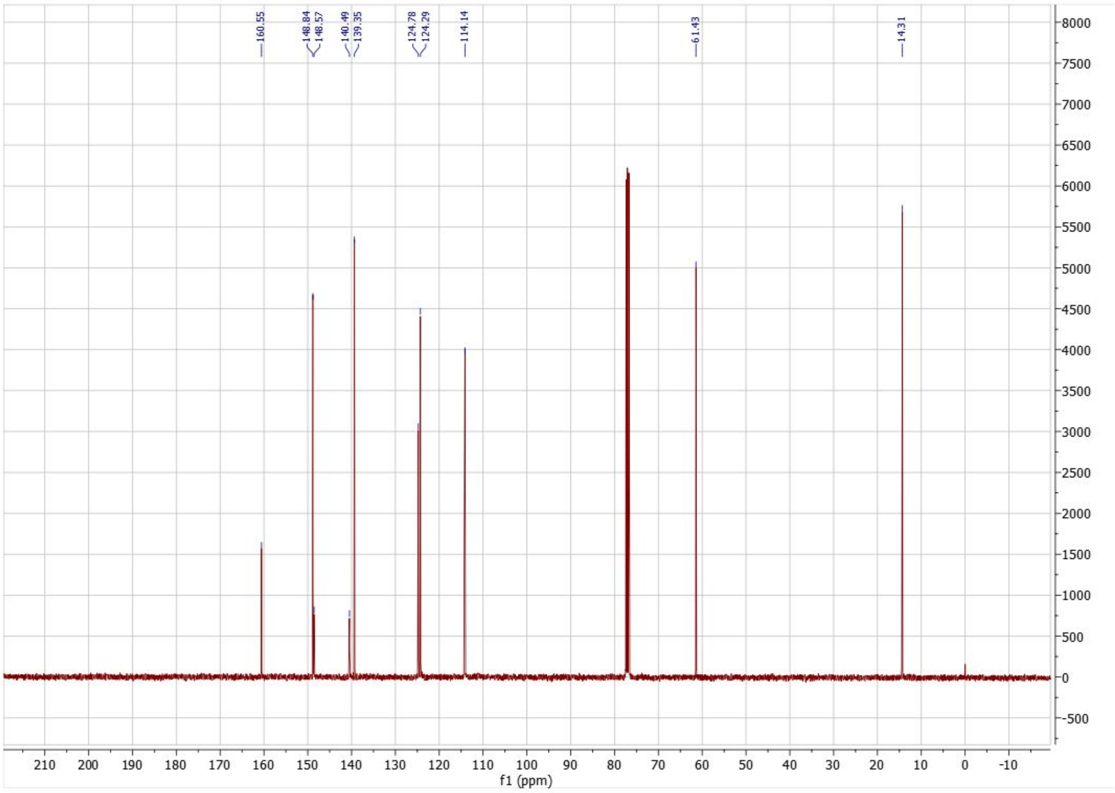

- ^{1}H NMR (400 MHz, $CDCl_3$) of ethyl 1-(3-methoxypyridin-2-yl)-1H-1,2,3-triazole-4-carboxylate (3b)

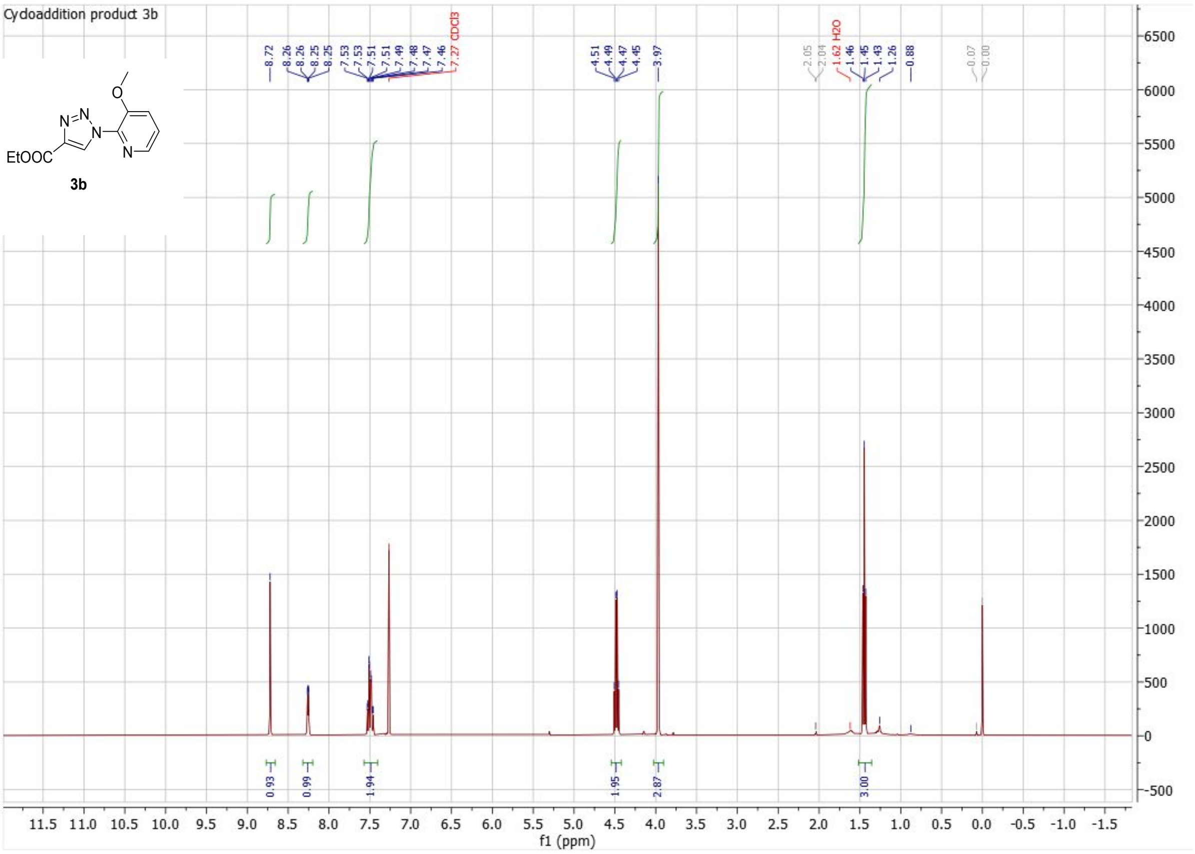

- ^{13}C NMR (101 MHz, $CDCl_3$) of ethyl 1-(3-methoxypyridin-2-yl)-1H-1,2,3-triazole-4-carboxylate (3b)

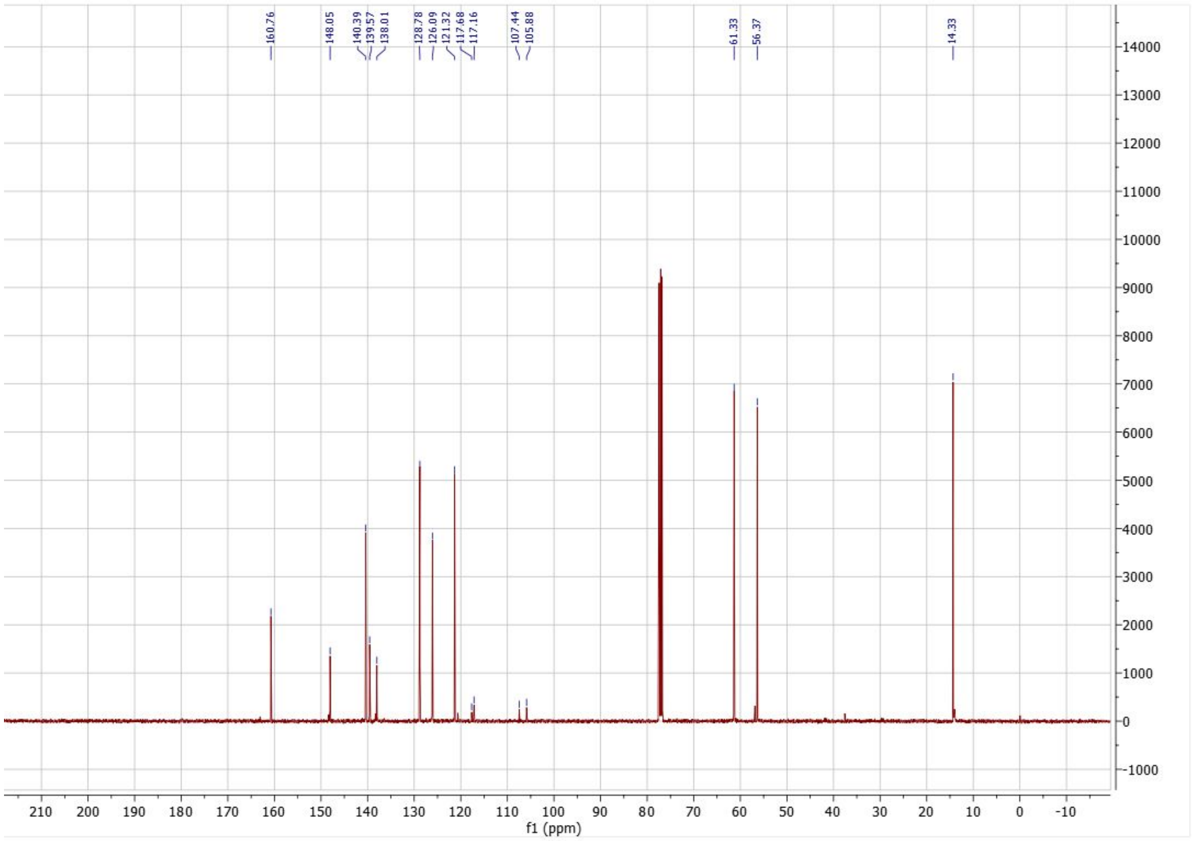

- ^{1}H NMR (400 MHz, $CDCl_3$) of ethyl 1-(5-methylpyridin-2-yl)-1H-1,2,3-triazole-4-carboxylate (3c)

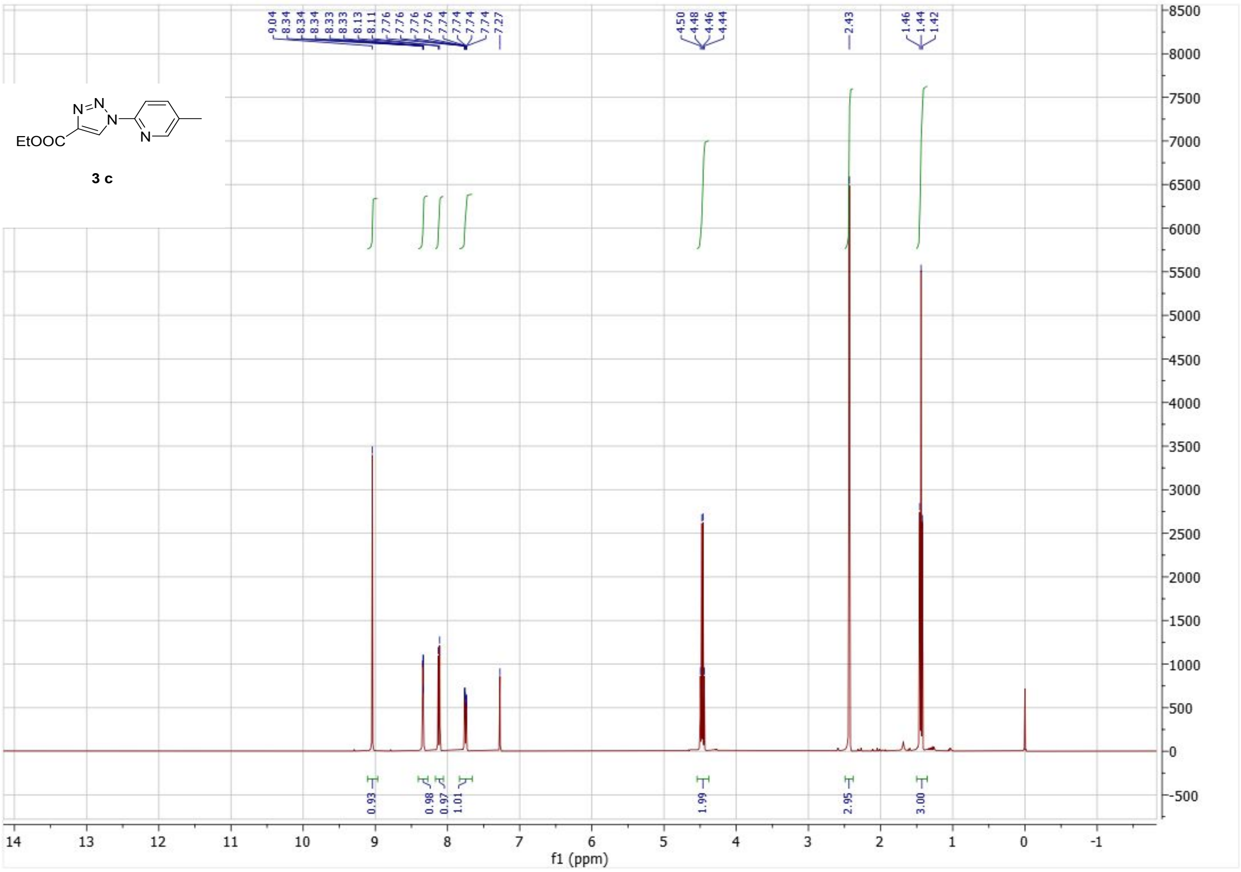

- ^{13}C NMR (101 MHz, $CDCl_3$) of ethyl 1-(5-methylpyridin-2-yl)-1H-1,2,3-triazole-4-carboxylate (3c)

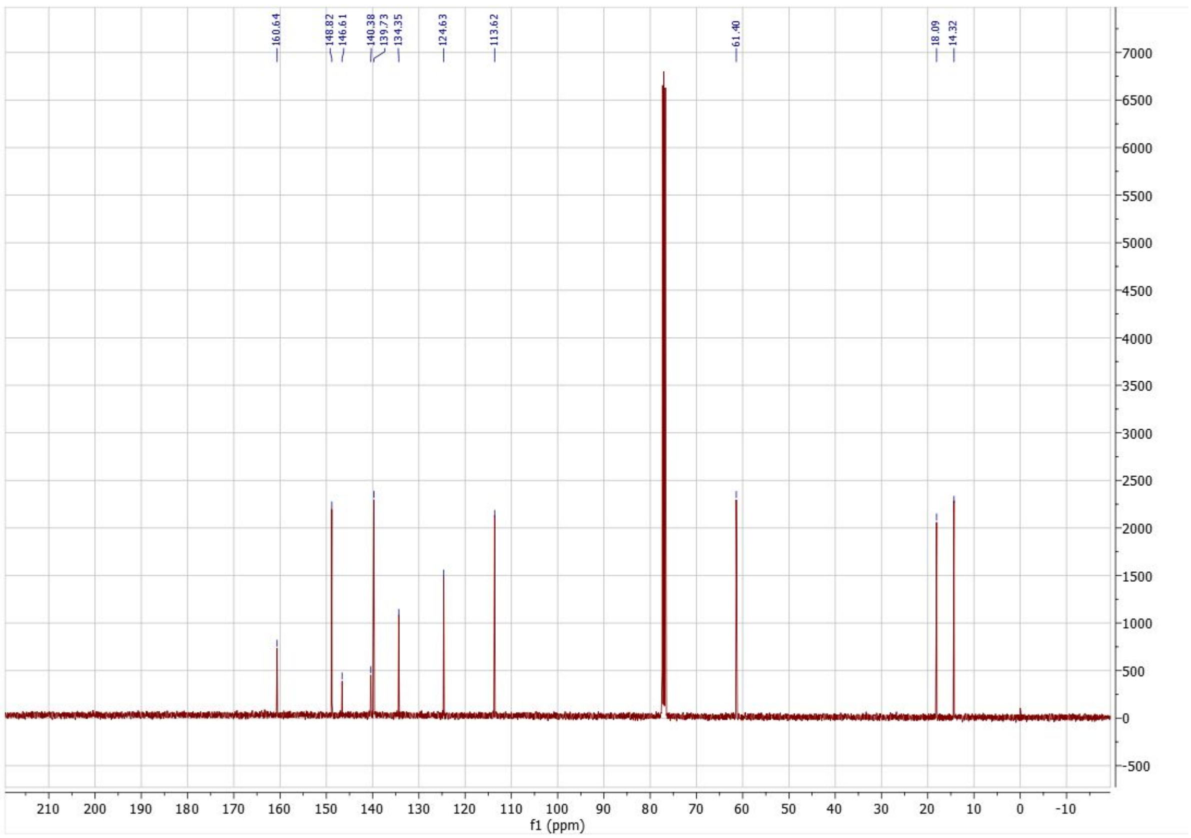

- ^{1}H NMR (400 MHz, $CDCl_3$) of ethyl 1-(5-fluoropyridin-2-yl)-1H-1,2,3-triazole-4-carboxylate (3d)

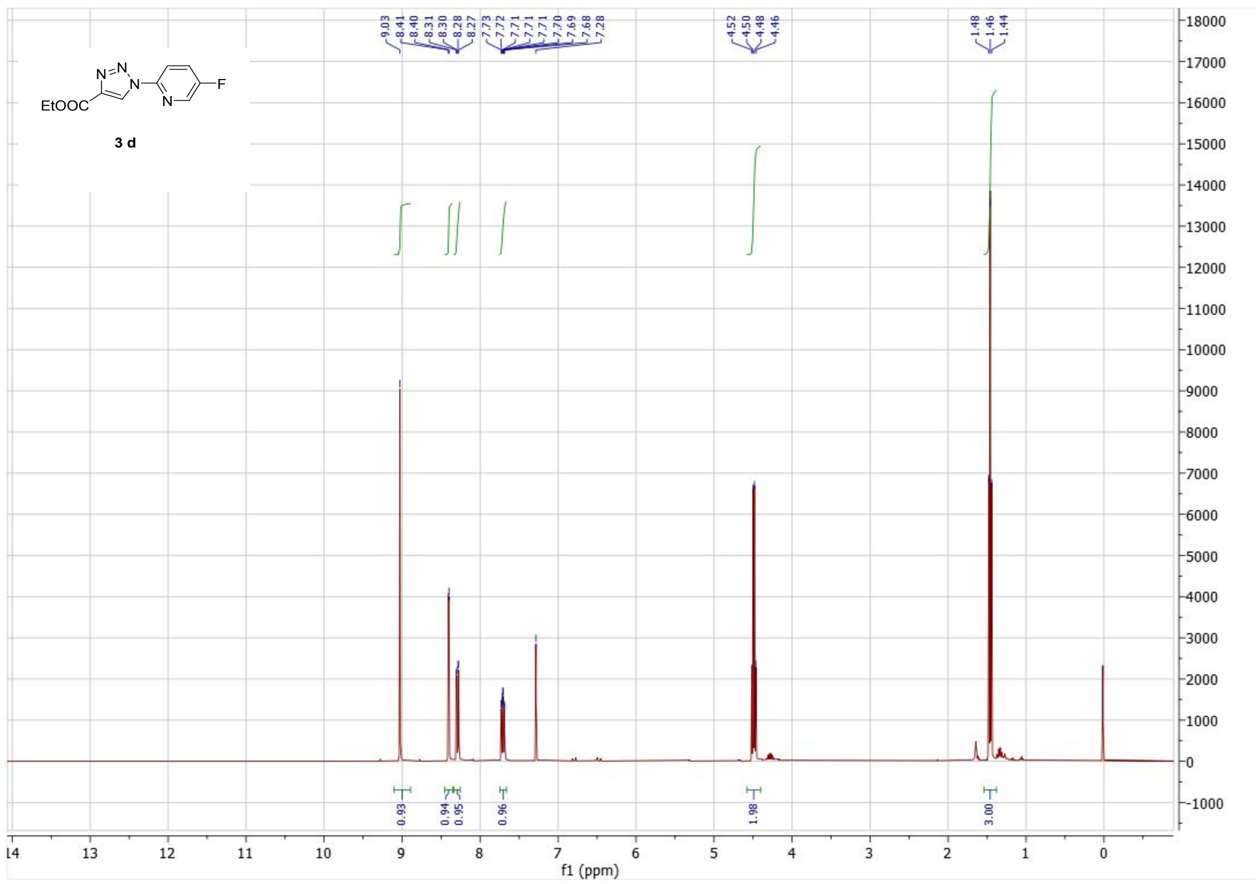

- ^{13}C NMR (101 MHz, $CDCl_3$) of ethyl 1-(5-fluoropyridin-2-yl)-1H-1,2,3-triazole-4-carboxylate (3d)

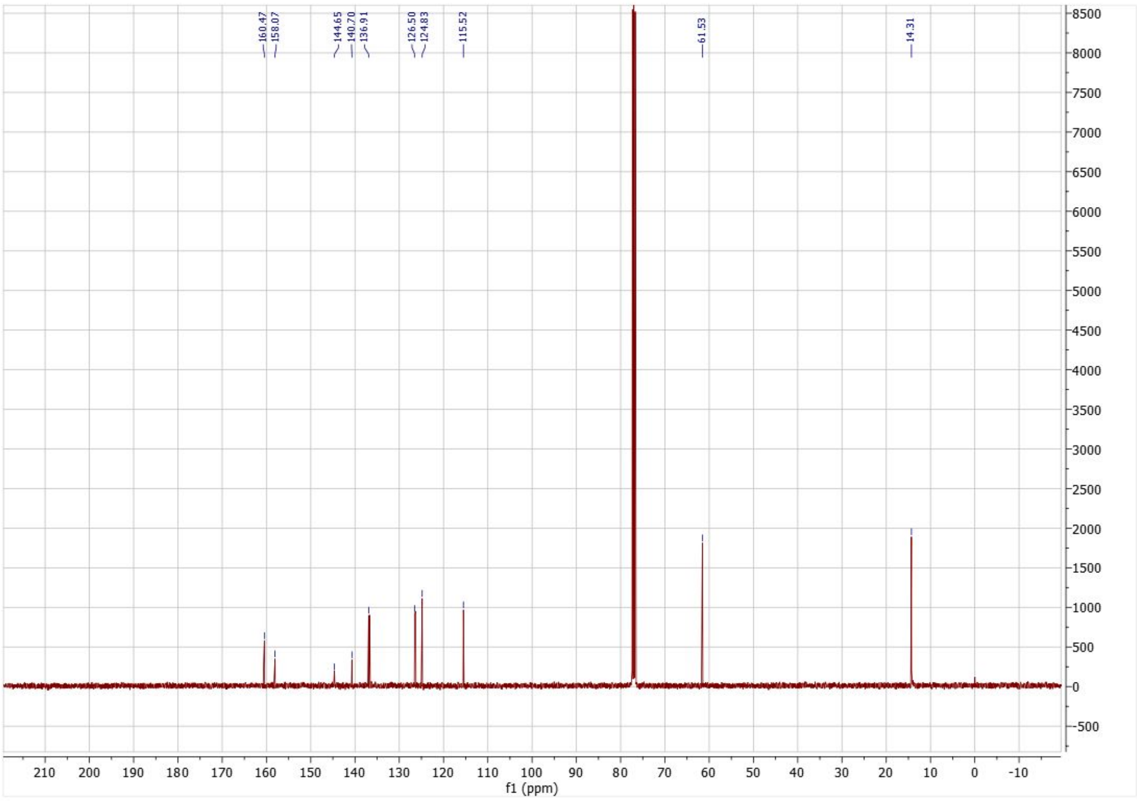

- ^{19}F NMR (376 MHz, $CDCl_3$) of ethyl 1-(5-fluoropyridin-2-yl)-1H-1,2,3-triazole-4-carboxylate (3d)

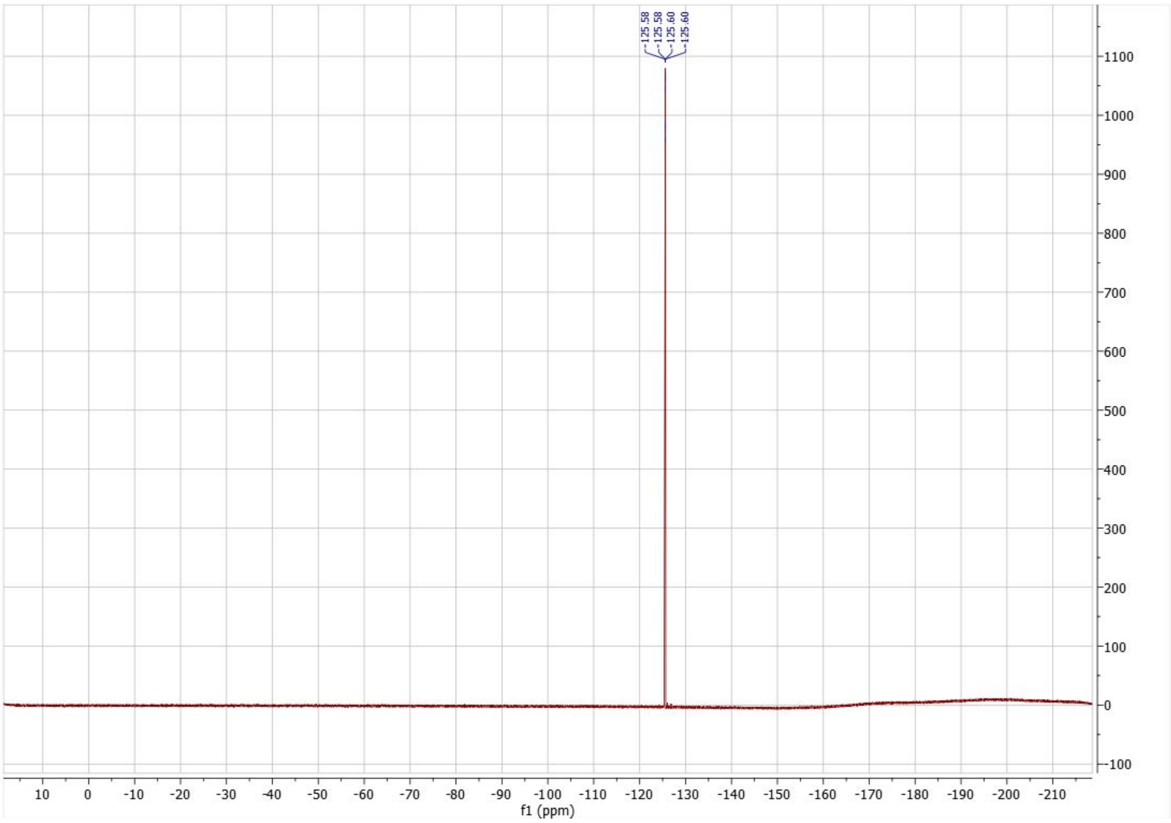

- ^{1}H NMR (400 MHz, $CDCl_3$) of ethyl 5-(benzylamino)-1-(pyridin-2-yl)-1H-1,2,3-triazole-4-carboxylate (5a)

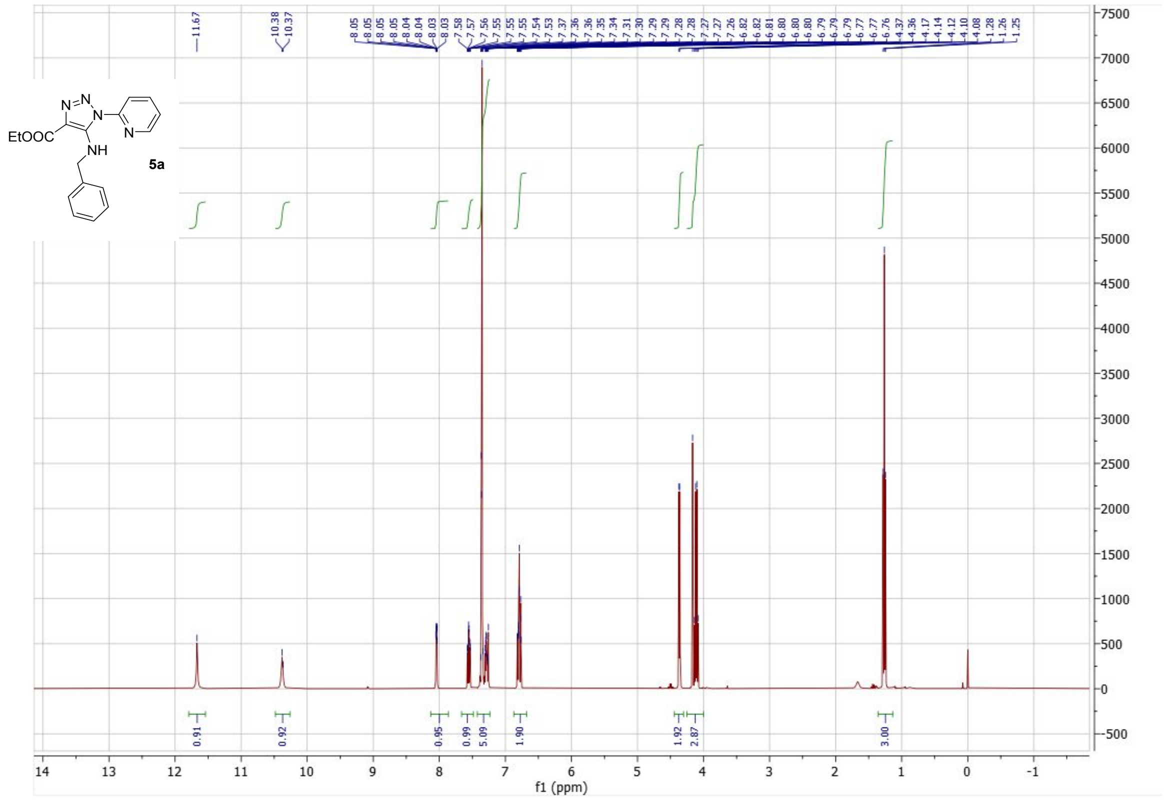

- ^{13}C NMR (101 MHz, $CDCl_3$) of ethyl 5-(benzylamino)-1-(pyridin-2-yl)-1H-1,2,3-triazole-4-carboxylate (5a)

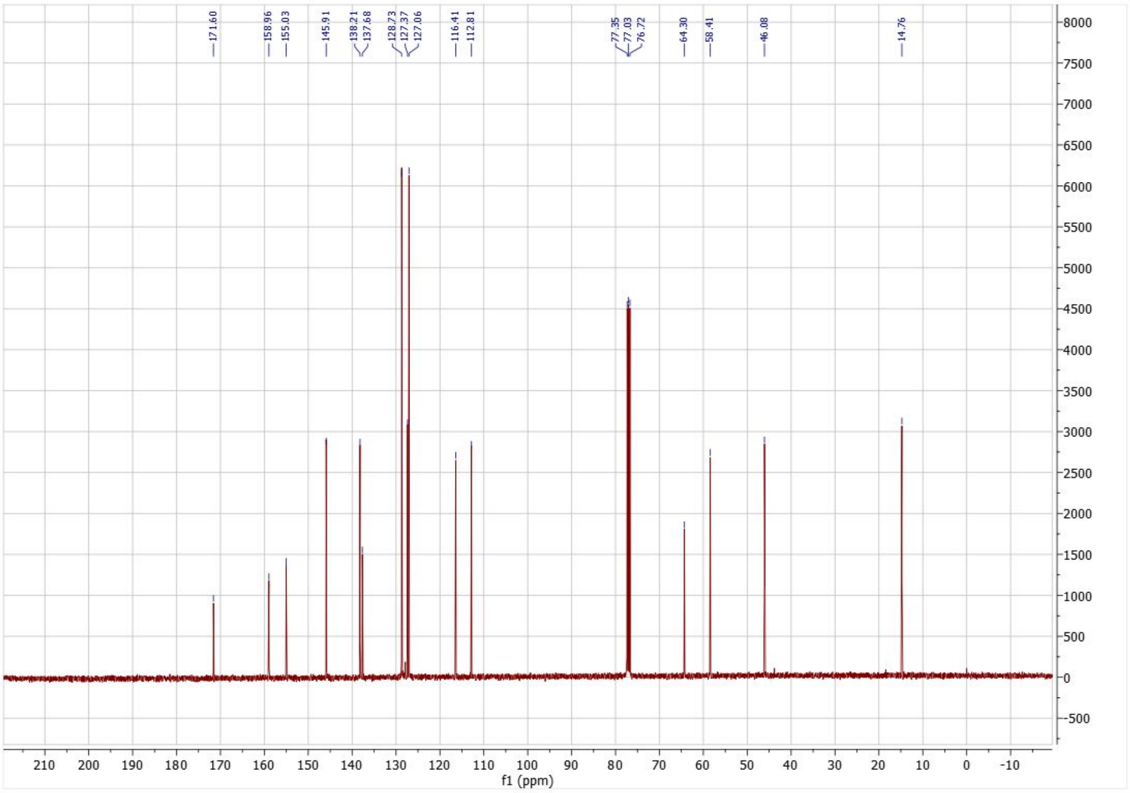

- ^{1}H NMR (400 MHz, $CDCl_3$) of ethyl-5-(benzylamino)-1-(3-methoxypyridin-2-yl)-1H-1,2,3-triazole 4 carboxylate (5b)

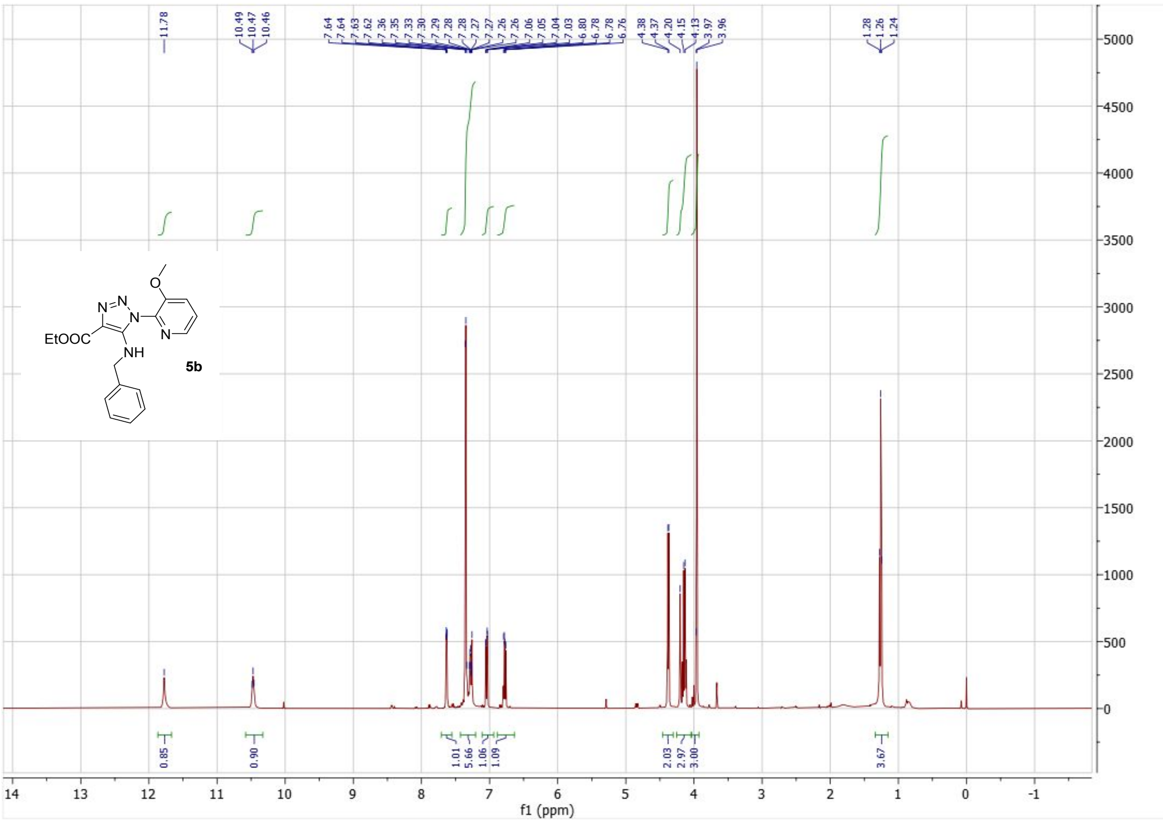

- ^{13}C NMR (101 MHz, $CDCl_3$) of ethyl-5-(benzylamino)-1-(3-methoxypyridin-2-yl)-1H-1,2,3-triazole 4 carboxylate (5b)

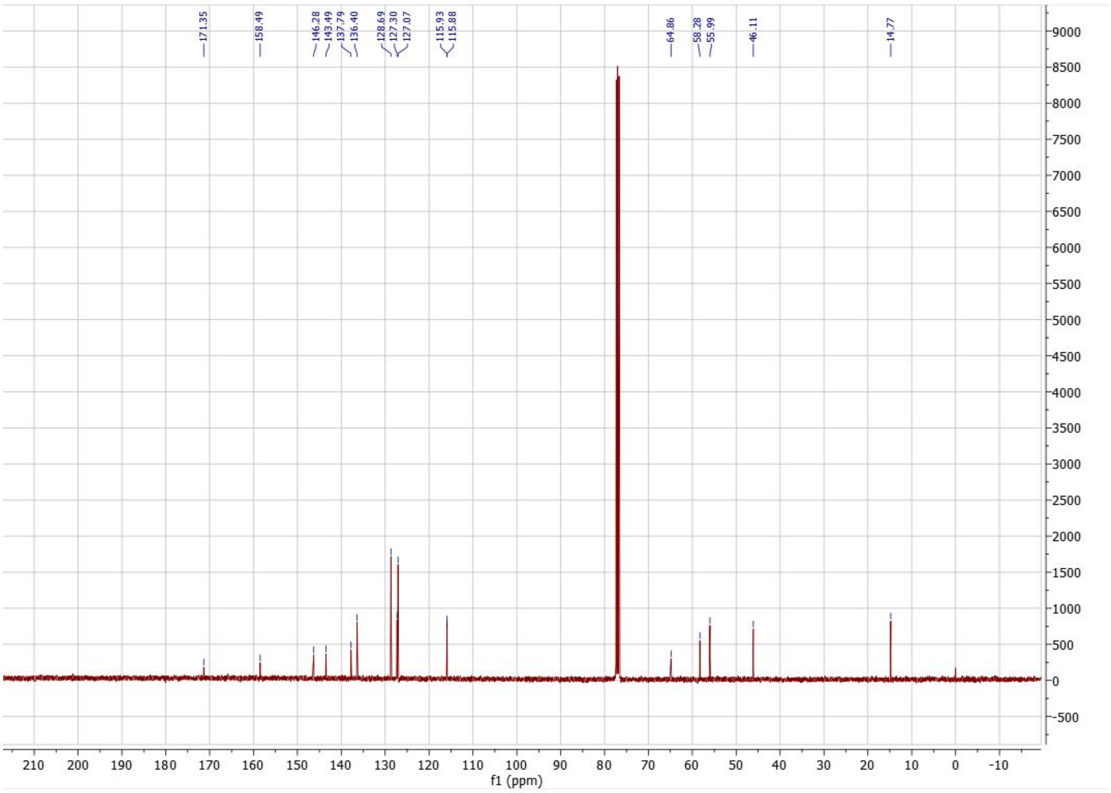

- ^{1}H NMR (400 MHz, $CDCl_3$) of ethyl 5-(heptylamino)-1-(3-methoxypyridin-2-yl)-1H-1,2,3-triazole-4-carboxylate (5c)

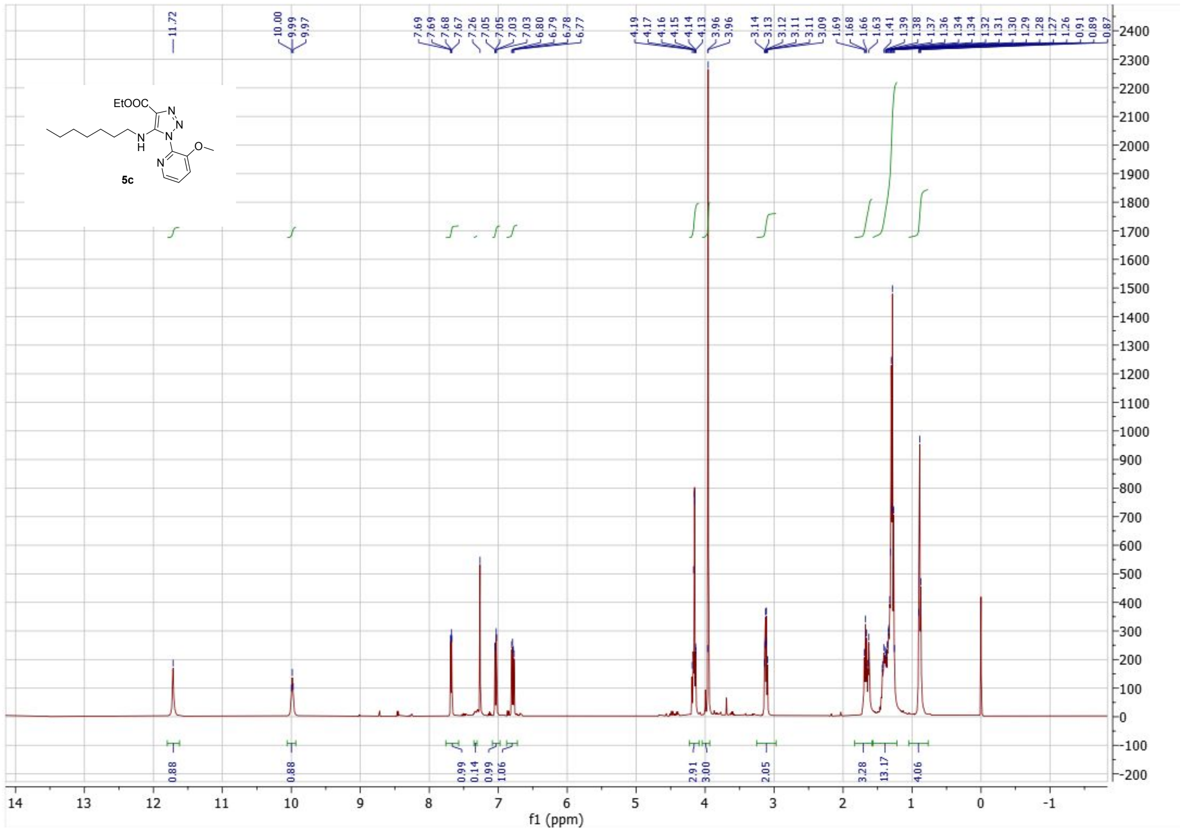

- ^{13}C NMR (101 MHz, $CDCl_3$) of ethyl 5-(heptylamino)-1-(3-methoxypyridin-2-yl)-1H-1,2,3-triazole-4-carboxylate (5c)

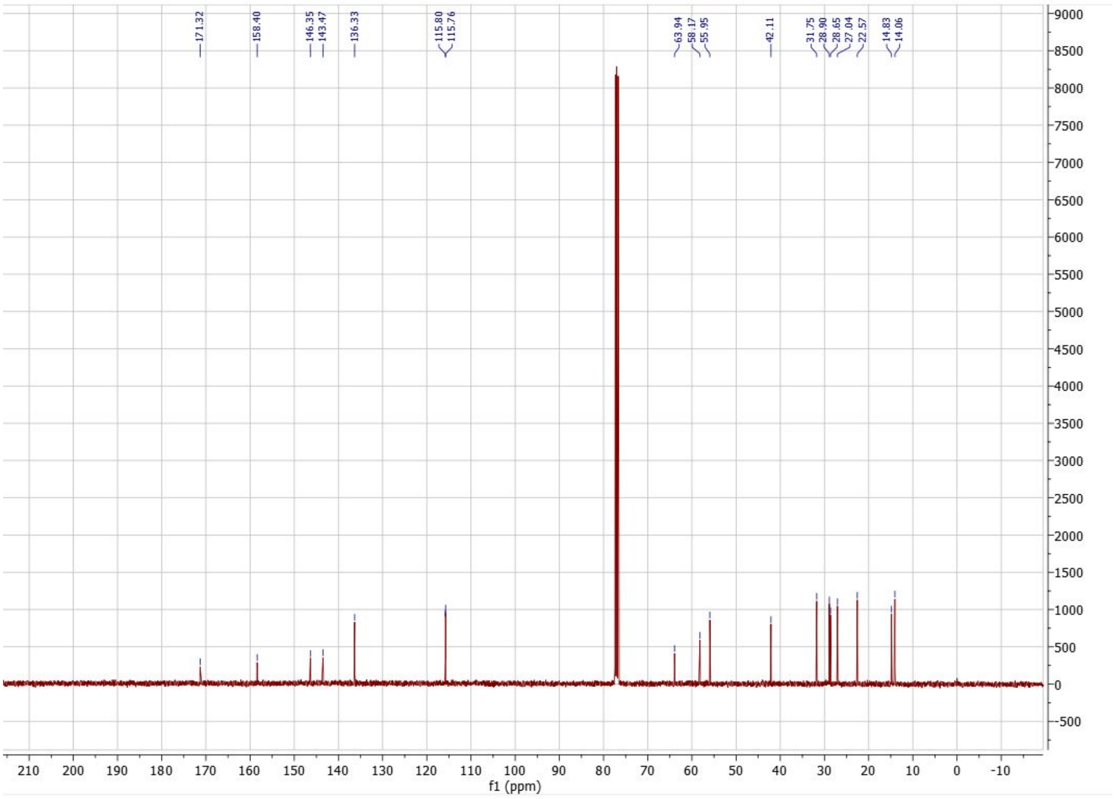

- ^{1}H NMR (300 MHz, $CDCl_3$) of ethyl-5-(butylamino)-1-(3-methoxypyridin-2-yl)-1H-1,2,3-triazole-4-carboxylate (5d)

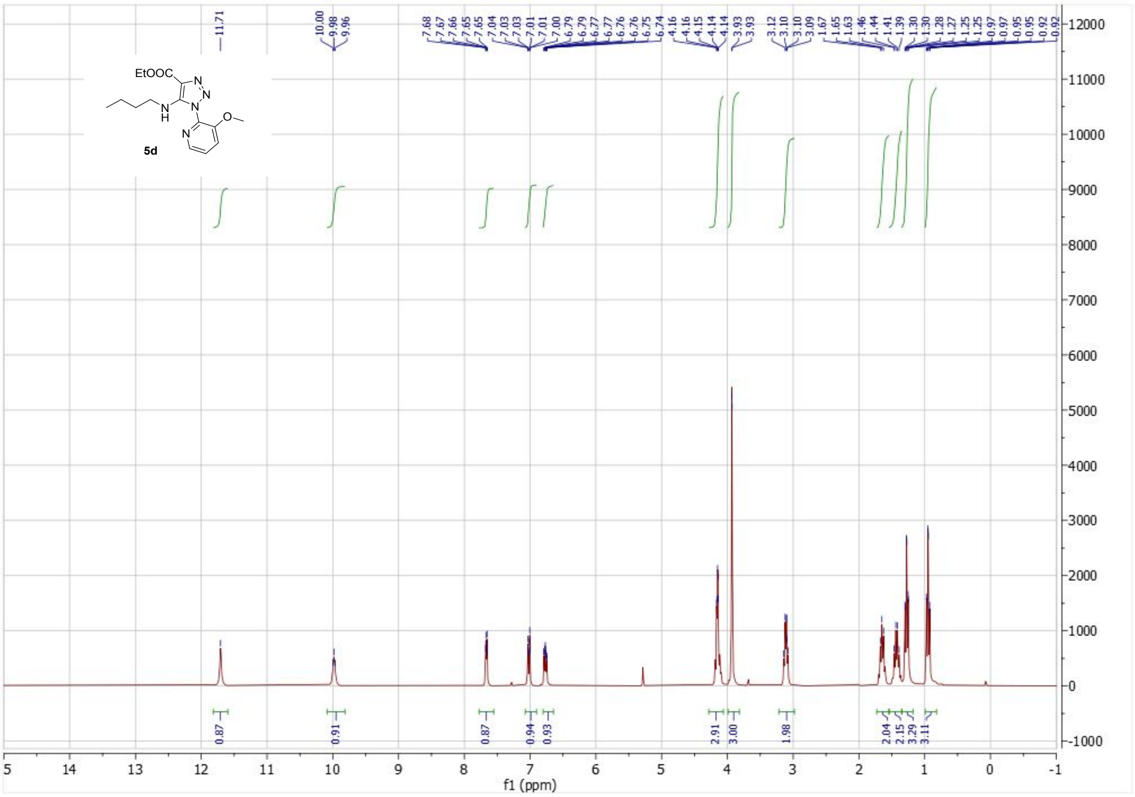

- ^{13}C NMR (75 MHz, $CDCl_3$) of ethyl-5-(butylamino)-1-(3-methoxypyridin-2-yl)-1H-1,2,3-triazole-4-carboxylate (5d)

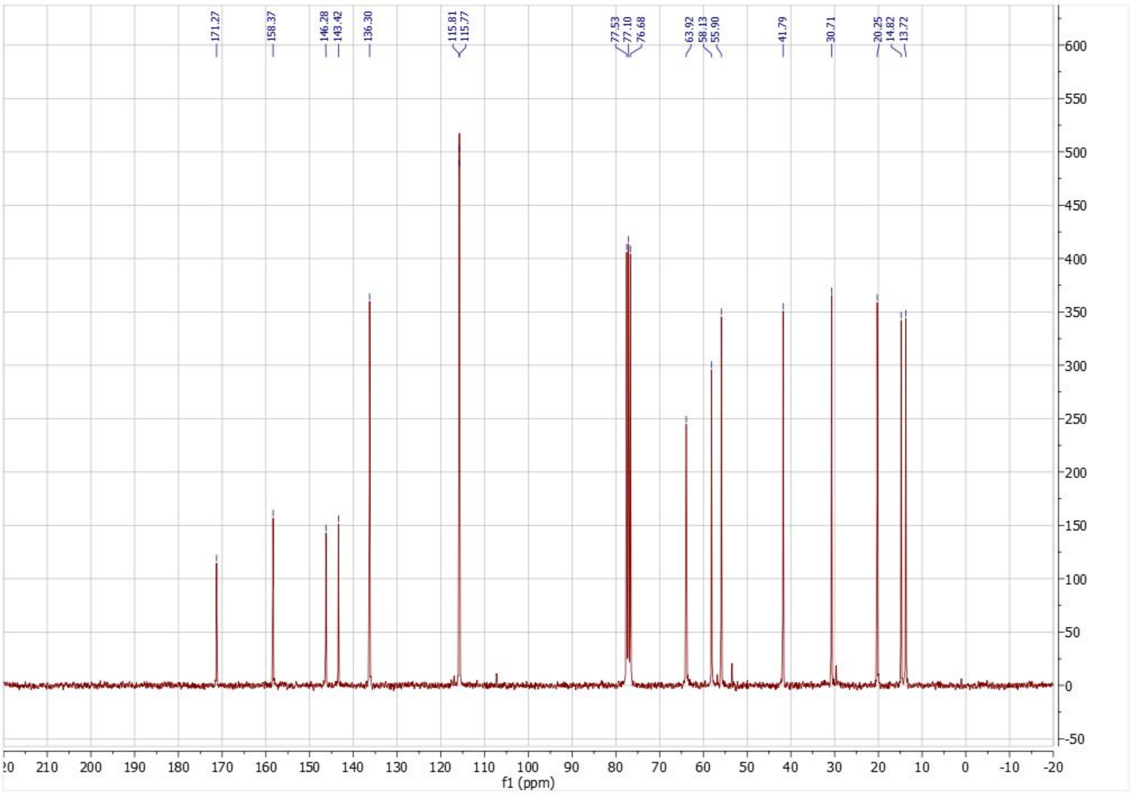

- **^{1}H NMR (300 MHz, $CDCl_3$) of ethyl-5-(benzylamino)-1-(5-fluoropyridin-2-yl)-1H-1,2,3-triazole-4-carboxylate (5e)**

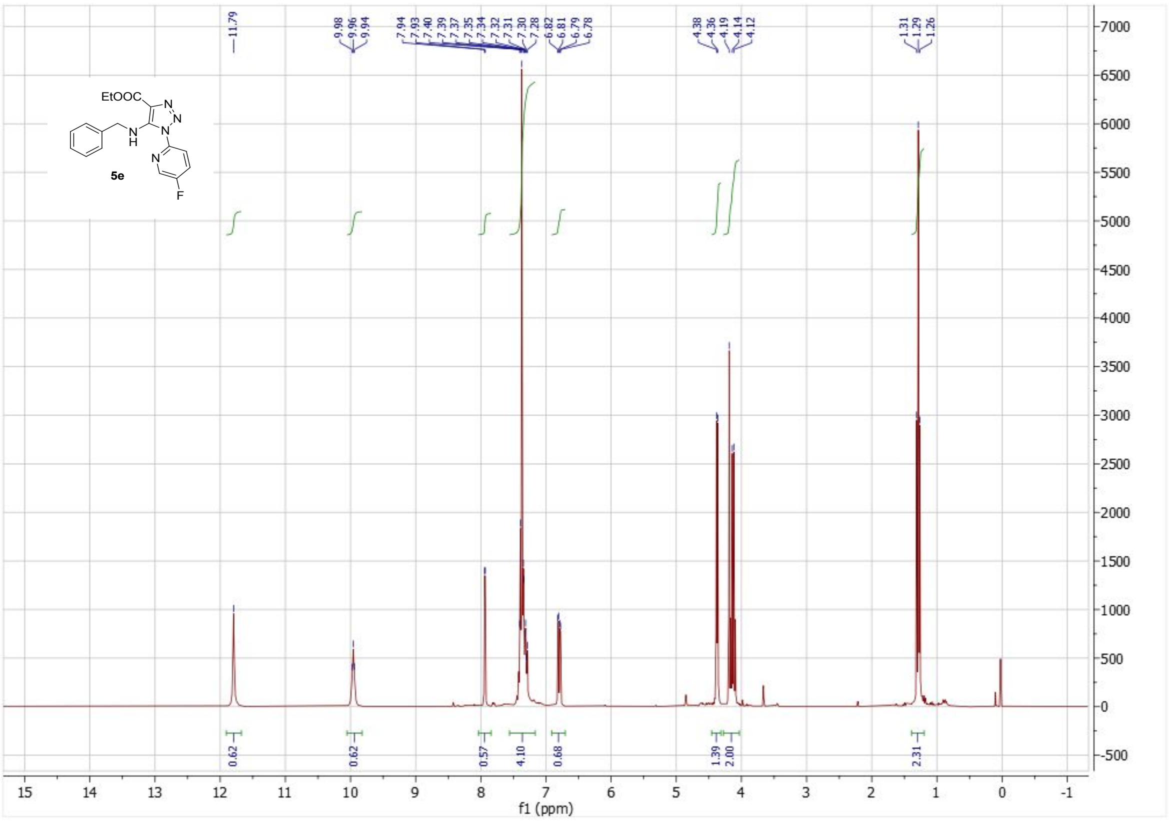

- **^{13}C NMR (75 MHz, $CDCl_3$) of ethyl-5-(benzylamino)-1-(5-fluoropyridin-2-yl)-1H-1,2,3-triazole-4-carboxylate (5e)**

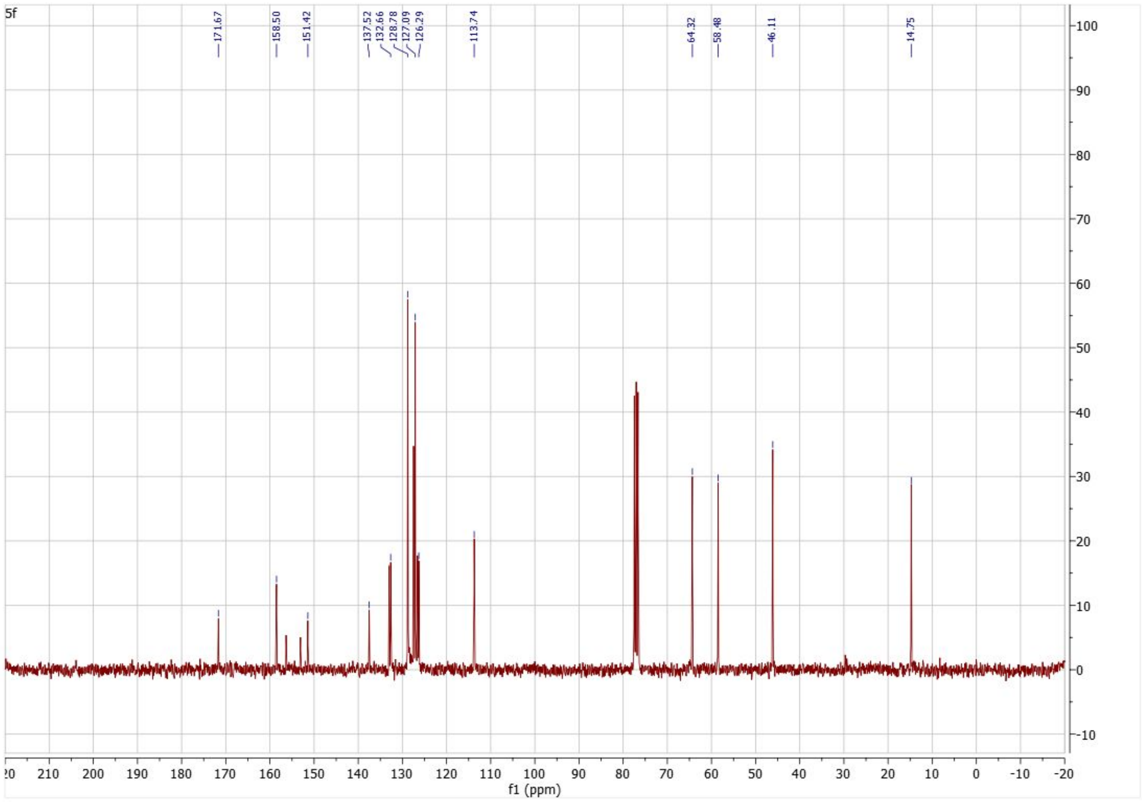

- **^{19}F NMR (276 MHz, $CDCl_3$) of ethyl-5-(benzylamino)-1-(5-fluoropyridin-2-yl)-1H-1,2,3-triazole-4-carboxylate (5e)**

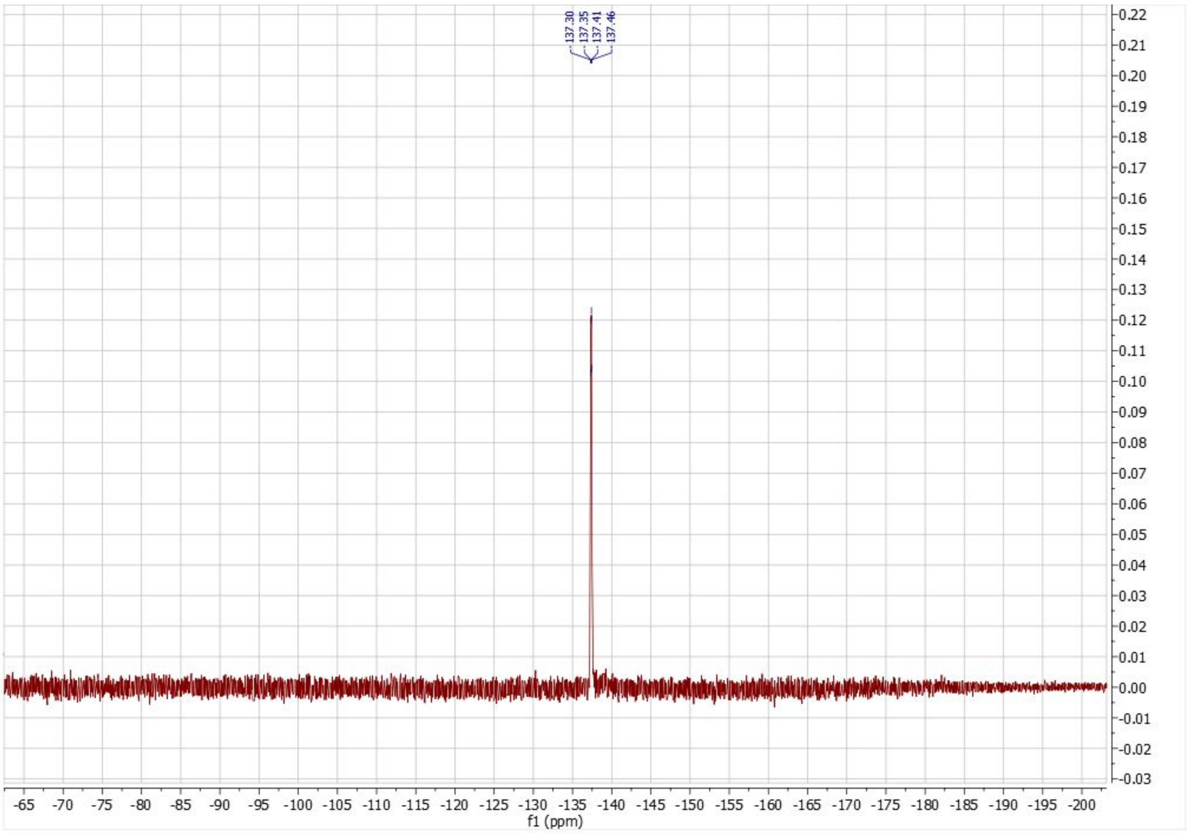

- **^{1}H NMR (300 MHz, $CDCl_3$) of ethyl-5-(butylamino)-1-(5-fluoropyridin-2-yl)-1H-1,2,3-triazole-4-carboxylate (5f)**

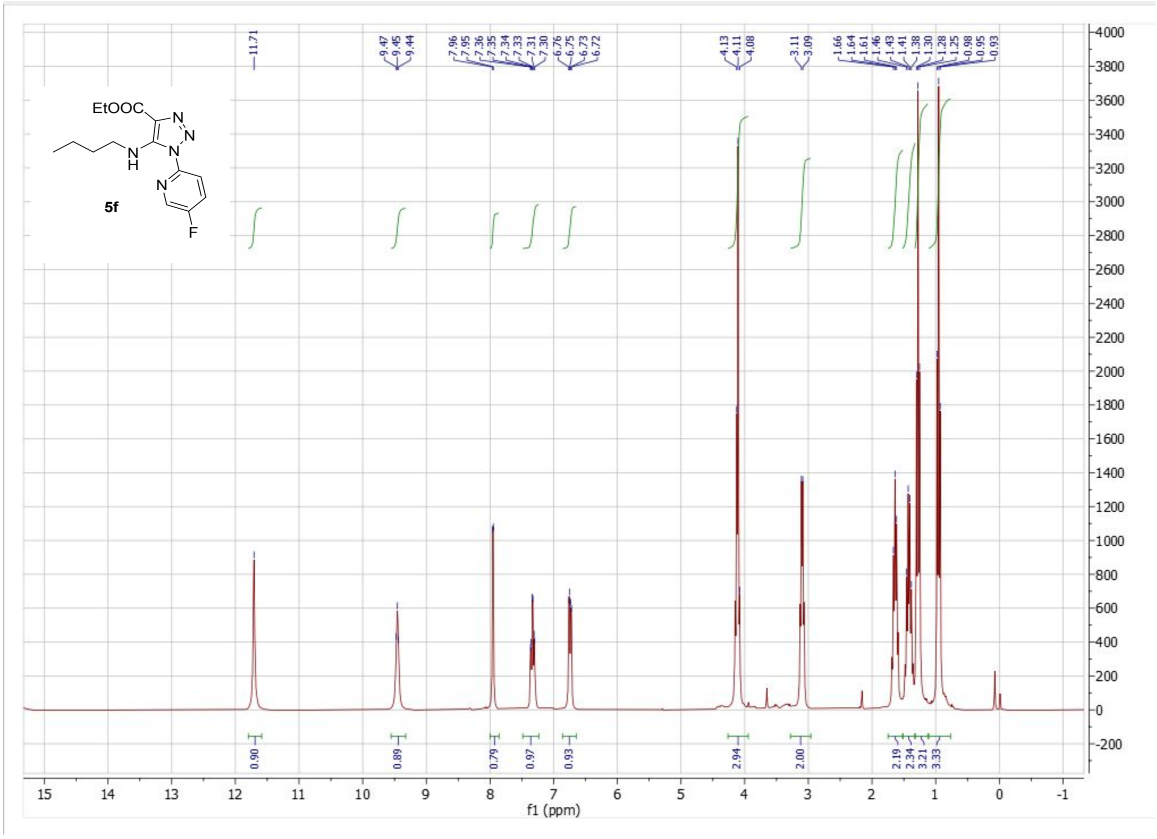

- **^{13}C NMR (75 MHz, $CDCl_3$) of ethyl-5-(butylamino)-1-(5-fluoropyridin-2-yl)-1H-1,2,3-triazole-4-carboxylate (5f)**

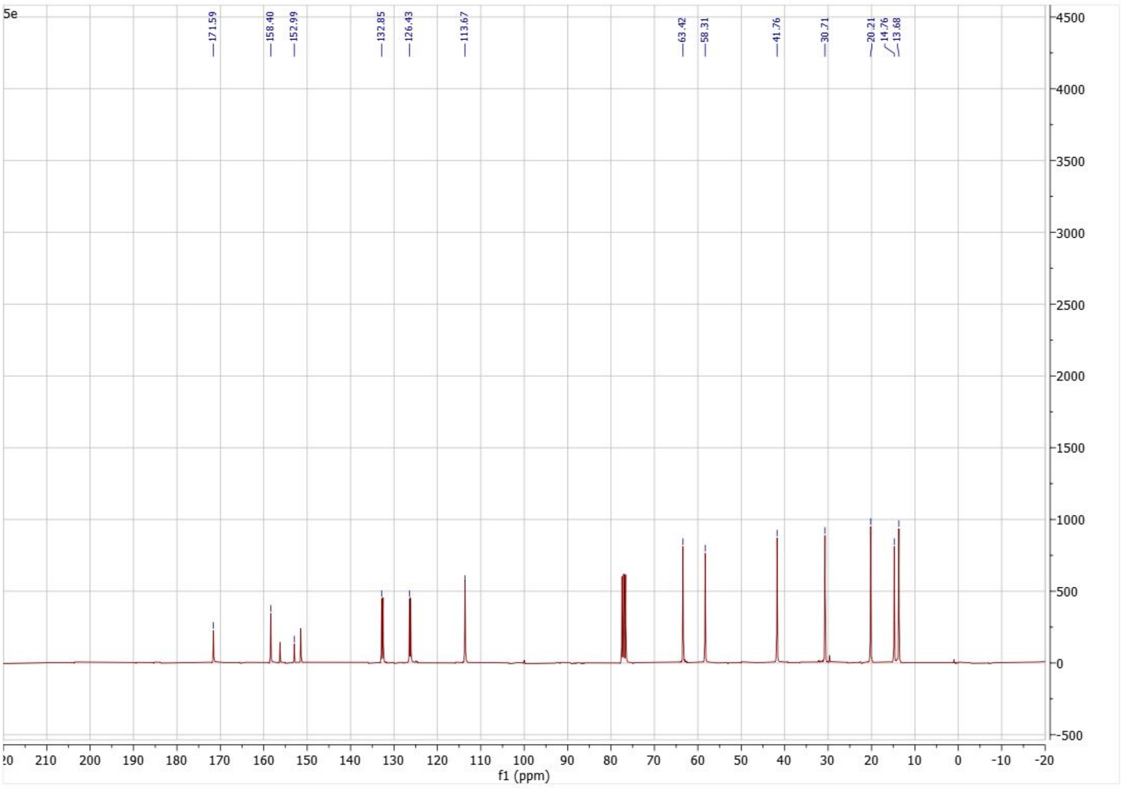

- **^{19}F NMR (276 MHz, $CDCl_3$) of ethyl-5-(butylamino)-1-(5-fluoropyridin-2-yl)-1H-1,2,3-triazole 4 carboxylate (5f)**

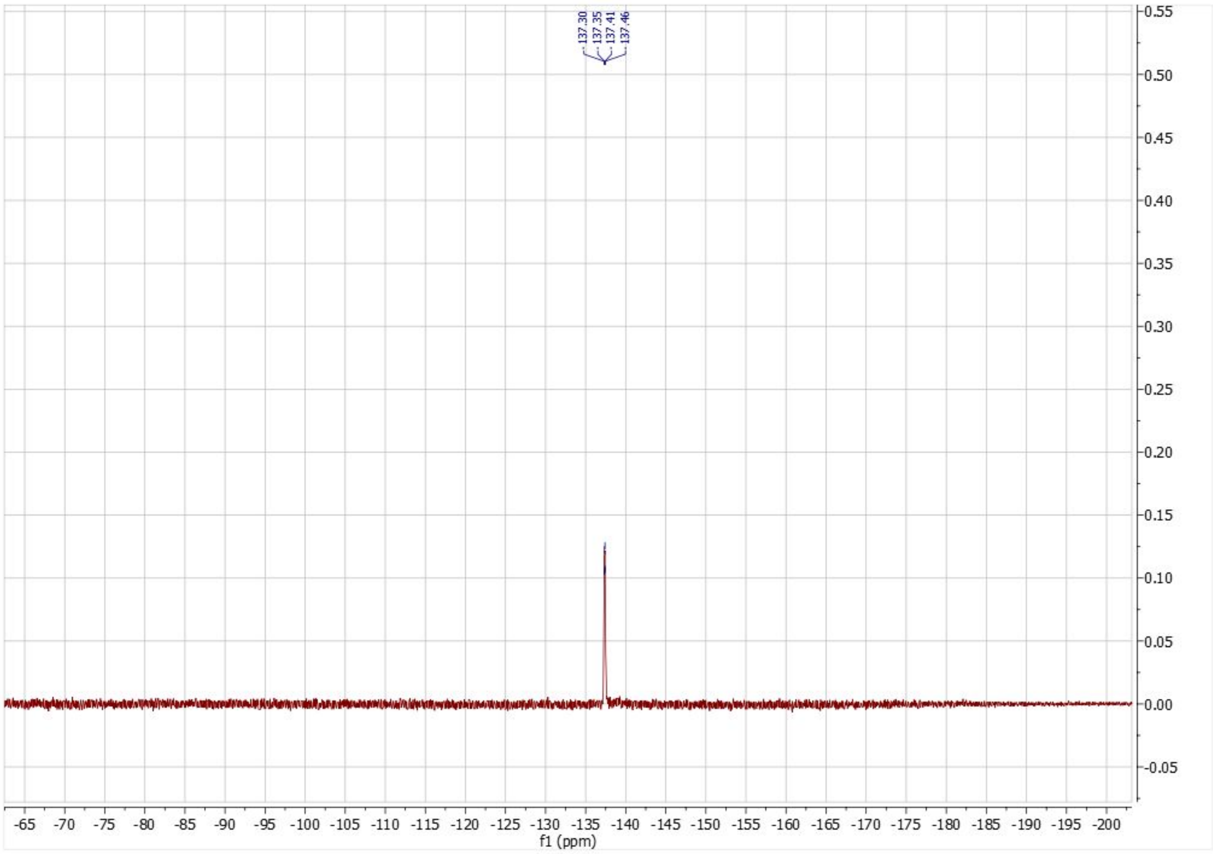

- ^{1}H NMR (300 MHz, $CDCl_3$) of ethyl 5-(benzylamino)-1-(5-methylpyridin-2-yl)-1H-1,2,3-triazole-4-carboxylate (5g)

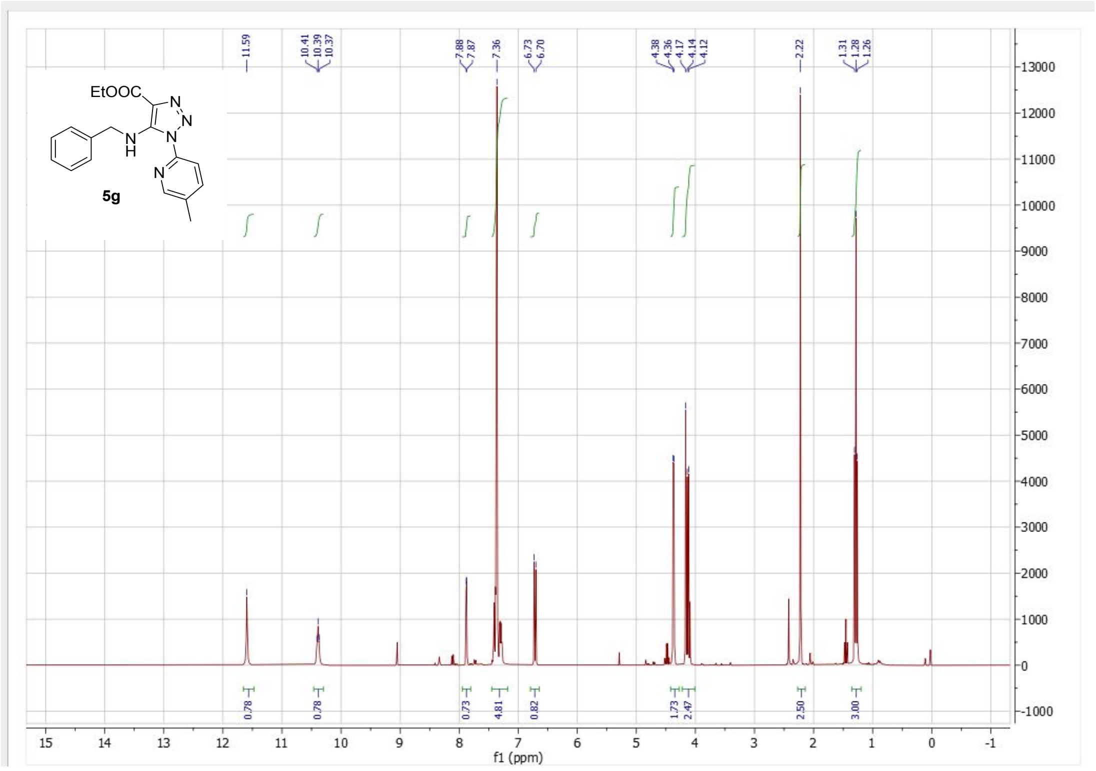

- ^{13}C NMR (75 MHz, $CDCl_3$) of ethyl 5-(benzylamino)-1-(5-methylpyridin-2-yl)-1H-1,2,3-triazole-4-carboxylate (5g)

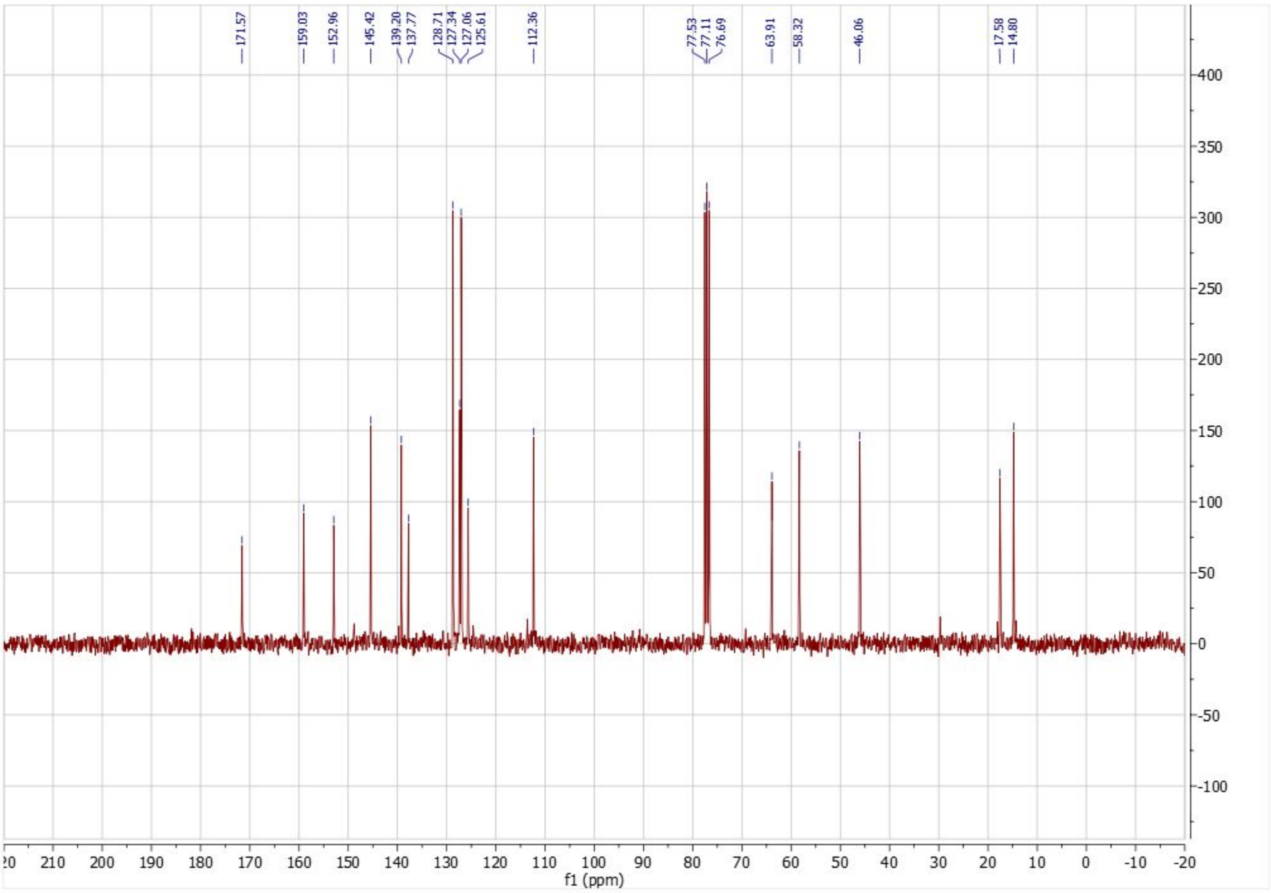
171.57
159.03
152.96
145.42
139.20
137.77
128.71
127.34
127.06
125.61
112.36
77.53
77.11
76.69
63.91
58.32
46.06
17.58
14.80
f1 (ppm)
